# A Comparative Study of Network-Based Machine Learning Approaches for Binary Classification in Metabolomics

**DOI:** 10.3390/metabo15030174

**Published:** 2025-03-03

**Authors:** Hunter Dlugas, Seongho Kim

**Affiliations:** 1 Biostatistics and Bioinformatics Core, Karmanos Cancer Institute, Detroit, MI 48201, USA; 2 Department of Oncology, Wayne State University School of Medicine, Detroit, MI 48201, USA

**Keywords:** artificial neural network, Bayesian neural network, binary classification, convolutional neural network, deep learning, Kolmogorov-Arnold network, machine learning, metabolomics, oncology, spiking neural network

## Abstract

**Background/Objectives**: Metabolomics has recently emerged as a key tool in the biological sciences, offering insights into metabolic pathways and processes. Over the last decade, network-based machine learning approaches have gained significant popularity and application across various fields. While several studies have utilized metabolomics profiles for sample classification, many network-based machine learning approaches remain unexplored for metabolomic-based classification tasks. This study aims to compare the performance of various network-based machine learning approaches, including recently developed methods, in metabolomics-based classification. **Methods**: A standard data preprocessing procedure was applied to 17 metabolomic datasets, and Bayesian neural network (BNN), convolutional neural network (CNN), feedforward neural network (FNN), Kolmogorov-Arnold network (KAN), and spiking neural network (SNN) were evaluated on each dataset. The datasets varied widely in size, mass spectrometry method, and response variable. **Results**: With respect to AUC on test data, BNN, CNN, FNN, KAN, and SNN were the top-performing models in 4, 1, 5, 3, and 4 of the 17 datasets, respectively. Regarding F1-score, the top-performing models were BNN (3 datasets), CNN (3 datasets), FNN (4 datasets), KAN (4 datasets), and SNN (3 datasets). For accuracy, BNN, CNN, FNN, KAN, and SNN performed best in 4, 1, 4, 4, and 4 datasets, respectively. **Conclusions**: No network-based modeling approach consistently outperformed others across the metrics of AUC, F1-score, or accuracy. Our results indicate that while no single network-based modeling approach is superior for metabolomics-based classification tasks, BNN, KAN, and SNN may be underappreciated and underutilized relative to the more commonly used CNN and FNN.

## 1. Introduction

Cancer remains one of the leading causes of death in the United States [1]. Metabolomics, the study of small non-peptide metabolites (i.e., molecules no larger than 1500 Daltons) within the body, has significantly advanced our understanding of cancer metabolism, facilitated the identification of biomarkers specific to various cancer types, and contributed to the development of novel cancer therapies [2,3].

**Figure 1:**
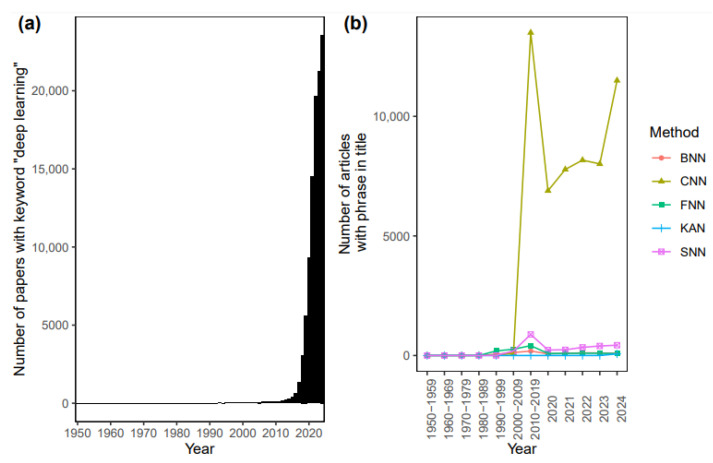
Growth in citations for deep learning and network-based machine learning models. (**a**) Number of citations with the keyword “deep learning” from 1950 to 2024 queried via PubMed on 27 January 2025. (**b**) Number of citations for each of the five types of network-based machine learning models considered in this study queried via Google Scholar on 27 January 2025. BNN: Bayesian neural network; CNN: convolutional neural network; FNN: feedforward neural network; KAN: Kolmogorov–Arnold network; SNN: spiking neural network.

The first network-based machine learning model implemented on a computer, ADALINE, was introduced in 1960 [4,5]. Interest in network-based modeling has grown steadily since the 1960s, with a surge of interest and novel applications beginning in the early 2000s, as indicated in Figure 1. This increase was likely driven by advancements in data storage capacity, improved algorithms, and the widespread availability of hardware capable of faster matrix multiplication. Today, machine learning is used to guide decisions and gain insights in nearly every conceivable industry, including oncology and metabolomics. In particular, machine learning has been applied to classify sample characteristics based on their metabolomic profile [6,7,8,9,10,11,12].

There are a variety of network-based machine learning approaches capable of classifying independent observations. Feedforward neural networks (FNNs) are the prototypical example of deep neural networks and have been applied to a wide range of classification and regression tasks in numerous fields. For example, FNNs have been utilized in civil engineering, genomics, and cybersecurity, among many other domains [13,14,15]. Convolutional neural networks (CNNs), a variant of FNNs, particularly tend to perform well when observations are tensors of rank greater than one (e.g., image classification, image segmentation, computer vision) [16,17,18,19,20]. This performance advantage is likely due to the convolution operation capturing spatial relationships across multiple dimensions. For instance, in image classification, the convolution operation considers information in a neighborhood of a given pixel rather than just the information in the given pixel.

Some recently developed network-based machine learning models have been specifically designed for particular problems and have been proposed to outperform others for certain tasks. For example, Bayesian neural networks (BNNs) offer improved uncertainty estimation, especially with respect to distinguishing between uncertainty due to noise (aleatoric) and uncertainty due to lack of knowledge (epistemic), which can help mitigate overfitting in small datasets. Over the past few years, BNNs have been applied to diverse fields including healthcare, image classification, pharmacology, electrical engineering, geology, and genomics [21,22,23,24,25,26,27]. Kolmogorov-Arnold networks (KANs) have been proposed to outperform FNNs in function fitting by their inventors [28]. Within months of their construction, KANs have been applied to a myriad of tasks such as gesture recognition, fault detection in bearings, mortality risk estimation, approximating solutions to partial differential equations, early gastric cancer diagnosis, time series forecasting, and computer vision [29,30,31,32,33,34,35,36]. Spiking neural networks (SNNs) have also gained traction with applications in fields such as robotics, electroencephalogram analysis, image classification, and chemistry [37,38,39,40,41]. Additionally, SNNs show promise in reducing the memory and energy consumption associated with more commonly used neural network models [42].

The high dimensionality of metabolomics data makes network-based machine learning models a natural choice for analysis. Both FNNs and CNNs have been applied to various aspects of metabolomics data analysis, including compound identification [43,44,45], peak alignment [46,47,48], peak detection [49,50], and classification based on metabolite abundance [8,9,10,11,12]. In several reviews of deep learning applied to metabolomics, FNNs and CNNs were the only type of artificial neural network considered [51,52]. However, to our knowledge, recently developed models such as BNNs, KANs, and SNNs have not yet been evaluated in the context of metabolomics-based classification tasks. The objective of this study is to compare these newer network-based machine learning models (i.e., BNNs, KANs, and SNNs) to the more commonly used FNNs and CNNs for metabolomics-based classification tasks.

## 2. Materials and Methods

### 2.1. Overview

**Figure 2:**
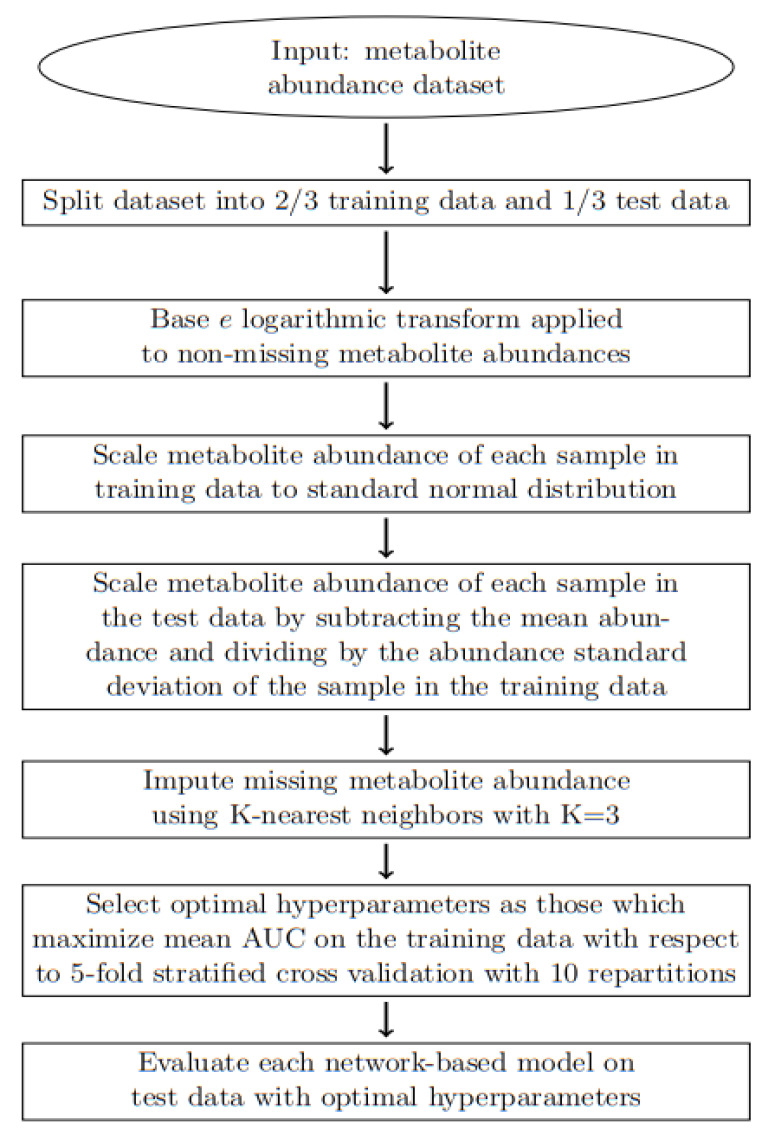
Flowchart of workflow.

An overview of our workflow is depicted in Figure 2. The datasets considered include the 10 datasets utilized by Mendez et al. [6,7,53,54,55,56,57,58,59,60,61,62] in addition to the 7 datasets from the Metabolomics Workbench [63,64,65,66,67,68,69,70] meeting the following criteria:

- Homo sapiens species;
- Sample size larger than 200;
- Cancer-related studies;
- Amenable to cancer-related binary classification.

The characteristics of each dataset are summarized in Table 1.

### 2.2. Data Preprocessing

A preprocessing approach similar to that used by Mendez et al. was applied for consistency [6]. Ideally, each dataset would consist of a training dataset and an independent test dataset to mimic real-world scenarios. However, the publicly available metabolomics datasets suitable for binary classification used in this study come as single datasets. Therefore, each dataset was split into training data (2/3) and testing data (1/3). The natural logarithm (base e) transformation was applied to non-missing metabolite abundances to reduce the variance of metabolite abundance. Normalization has been shown to improve both performance and efficiency [71], and the metabolite abundance of each sample was scaled to approximate a standard normal distribution, similar to the recommendation of TensorFlow [72]. That is, for the training data, the metabolite abundance of each sample was scaled to have a mean of 0 and a variance of 1. For the test data, the metabolite abundance of each sample was scaled to approximate a standard normal distribution by subtracting the mean abundance and dividing by the abundance standard deviation of the given sample in the training data. Missing metabolite abundance values were imputed using K-nearest neighbors with K = 3 following the approach taken by Mendez et al. [6].

### 2.3. Network-Based Machine Learning Models

All neural networks described in this section are assumed to have one or more hidden layers.

#### 2.3.1. Feedforward Neural Networks (FNNs)

Feedforward neural networks (FNNs) do not have connections between nodes within the same layer. If such connections exist, then the network is referred to as *recurrent*, not *feedforward*. Note that recurrent neural networks are used for analyzing sequential data (e.g., speech or time series) and are thus not implemented in this study.

A, FNN is a function $f:R^{N}\to R^{M}$ represented as a composition:

(1) $$f:\phi_{L+1}\circ \sigma_{L}\circ \phi_{L-1}\circ \cdots \circ \sigma_{1}\circ \phi_{1}$$

where $\phi_{i}:R^{N_{i-1}}\to R^{N_{i}}$ is the affine transformation $\phi_{i}\left(x\right)=A_{i}x+b_{i}$ and $\sigma_{i}:\;R^{N_{i}}\to R^{N_{i}}$ is a mapping where each component is an activation function from R to R. Common choices for the activation function include the sigmoid activation function $f\left(x\right)=\frac{1}{1+e^{-x}}$ and the rectified linear unit (ReLU) activation function $f\left(x\right)=\max\left(0,x\right)$. The optimal weights (i.e., linear transformations $A_{i}$) and biases (i.e., translation vectors $b_{i}$) that define the FNN given a user-specified architecture are determined by minimizing a loss function, which is typically mean squared error or cross-entropy for regression or classification tasks, respectively. The chain rule from calculus is repeatedly applied to calculate the gradient of the loss function in a process known as backpropagation.

#### 2.3.2. Convolutional Neural Networks (CNNs)

A convolutional neural network (CNN) is a specialized type of FNN that includes one or more convolutional layers, typically combined with pooling layers and fully connected layers. A convolutional layer consists of a sliding window that slides along the nodes in a given layer, applying a pooling function at each location of the sliding window. The pooling function is typically the maximum or arithmetic mean. For example, in a one-dimensional convolution, if a given layer is represented as $\left(x_{1},x_{2},\ldots ,x_{n}\right)\in R^{n}$, a sliding window of length 3 with a maximum pooling function results in the next layer being $\left(max\left(x_{1},x_{2},x_{3}\right),max\left(x_{2},x_{3},x_{4}\right),\ldots ,max\left(x_{n-2},x_{n-1},x_{n}\right)\right)\in R^{n-2}$. This procedure generalizes to higher-dimensional tensors.

#### 2.3.3. Bayesian Neural Networks (BNNs)

Minimizing the loss function in a FNN, as described in Section 2.3.1, is similar to maximum likelihood estimation in the sense that a point estimate of the parameters (i.e., the weights and biases) is obtained through some optimization process. Sometimes FNNs—with their single point estimate of the weights and biases obtained from minimizing some loss function on training data—are overconfident in predictions on unseen data. To address this, Bayesian neural networks (BNNs), a family of stochastic artificial neural networks, use Bayesian inference to estimate the uncertainty associated with predictions [73].

If θ denotes the model parametrization (i.e., weights and biases) and D denotes the training data, then Bayes’s Theorm yields the posterior:

(2) $$p\left(\theta |D\right)=\frac{p\left(D|\theta \right)}{p\left(D\right)}p\left(\theta \right).$$

Estimating this posterior is often intractable largely due to the difficulty of computing $p\left(D\right)=\int_{\theta }p\left(D|\theta \right)p\left(\theta \right)d\theta$. Once the prior $p\left(\theta \right)$ is specified, either a Markov Chain Monte Carlo (MCMC) or variational inference approach can be employed to approximate Equation (2) [73].

#### 2.3.4. Spiking Neural Networks (SNNs)

Neural networks are named as such due to their similarity to biological neurons, with both representing nodes in a graph connected by edges. In artificial neural networks, an edge represents the weight of the connection, and in biological systems, it represents an electric current (spike). Of all types of neural networks, spiking neural networks (SNNs) most closely resemble biological neurons due to including a temporal component and accounting for the timing of spikes and their rate of decay.

In SNNs, activation functions are indicator functions $\phi_{spike,t^{⋆}}^{L^{⋆}}$ that attain values of 0 or 1 according to specific criteria. If the membrane potential:

(3) $$U_{\eta ,t}^{L}=\beta_{\eta }^{L}U_{\eta ,t-1}^{L}+W_{\eta }^{L}h_{t}^{L-1}-\phi_{spike,t-1}^{L}U_{threshold,\eta }^{L}$$

of the η-th neuron in the L-th layer at time t exceeds the membrane threshold $U_{threshold,\eta }^{L}$, a spike is recorded. Note that $W_{\eta }^{L}h_{t}^{L-1}$ denotes the product of the weight matrix and the vector of preceding layer values $h_{t}^{L-1}$. The constant $\beta_{\eta }^{L}$ is the membrane potential decay rate of the η-th neuron in the L-th layer. This model is known as the canonical leaky integrate and fire (LIF) model and can be realized as the forward Euler solution of an ordinary differential equation. For more details on the formulation of SNNs, see [74,75].

#### 2.3.5. Kolmogorov-Arnold Networks (KANs)

In FNNs, the machine learning practitioner sets the activation functions to be used, and an optimization procedure determines the optimal weights and biases (i.e., the edges between nodes in the network and the offsets of each layer). In contrast, Kolmogorov-Arnold networks (KANs) use an optimization procedure to learn optimal activation functions for each edge in the network [28]. These networks are motivated by the Kolmogorov-Arnold Representation Theorem which states that a continuous, multivariable, real-valued function $f:{\left[0,1\right]}^{n}\to R$ can be written as a linear combination of univariable continuous functions $\phi_{q,p}:\left[0,1\right]\to R$, $\Phi_{q}:R\to R$ given by $f\left(\left(x_{1},x_{2},\ldots ,x_{n}\right)\right)=\sum_{q=1}^{2n+1}\Phi_{q}\left(\sum_{p=1}^{n}\phi_{q,p}\left(x_{p}\right)\right)$ [28]. In practice, the univariable real-valued continuous functions $\phi_{q,p}$ and $\Phi_{q}$ are taken to be B-splines.

### 2.4. Training and Evaluation of Network-Based Machine Learning Models

The Adam optimizer was used for all five network-based machine learning models [76]. When performing K-fold cross-validation for model selection in classification tasks, it has been suggested that the train and test splits be stratified such that the same proportion of cases are present in each fold [77,78]. Therefore, for each dataset and model, 5-fold stratified cross-validation with 10 partitions (where each partition defines the 5 folds) was performed. Optimal hyperparameters were determined by maximizing the mean AUC across the 50 (=5 folds × 10 partitions) test folds within the training data. For each model, the grid search of hyperparameters were:

- BNN [79]:
  ◦ Number of epochs: {100, 200}
  ◦ Learning rate: {0.001, 0.01, 0.1}
  ◦ Number of neurons in single hidden layer: {0, 1, 2, 3, 4, 5, 10, 20}
  ◦ Activation function of each neuron in hidden layer: {sigmoid, ReLU}
  ◦ Weight of Kullback-Leibler divergence in loss function: {0.1, 0.01, 0.001}
- CNN [80]:
  ◦ Number of epochs: {100, 200}
  ◦ Learning rate: {0.001, 0.01, 0.1}
  ◦ Number of neurons in first hidden layer: {16, 32}
  ◦ Number of neurons in second hidden layer: {32, 64}
  ◦ Activation function of each neuron in hidden layers: {sigmoid, ReLU}
  ◦ Kernel size (i.e., size of convolution window): {2, 3, 4}
  ◦ Max pooling size: {2, 3}
  ◦ Dropout rate (i.e., fraction of input units to drop): {0.5}
- FNN [80]:
  ◦ Number of epochs: {100, 200}
  ◦ Learning rate: {0.001, 0.01, 0.1}
  ◦ Number of neurons in single hidden layer: {0, 1, 2, 3, 4, 5, 10, 20}
  ◦ Activation function of each neuron in hidden layer: {sigmoid, ReLU}
- KAN [28]:
  ◦ Number of epochs: {100, 200}
  ◦ Order of spline in each activation function: {2, 3}
  ◦ Number of grid intervals: {2, 3}
  ◦ Number of neurons in hidden layer: {0, 1, 2, 3, 4, 5, 10, 20}
- SNN [74,81,82]:
  ◦ Number of epochs: {100, 200}
  ◦ Learning rate: {0.001, 0.01, 0.1}
  ◦ Membrane potential decay rate: {0.5, 0.75, 1}
  ◦ Number of time steps: {10, 20}
  ◦ Number of neurons in single hidden layer: {1, 2, 3, 4, 5, 10, 20}
  ◦ Rate which target neuron is expected to fire: {0.8, 0.9}.

To minimize bias, the same data partitions were used across all models. Each model was evaluated on the test data using AUC, F1-score, and accuracy with the optimal hyperparameters.

## 3. Results

### 3.1. Evaluation with Respect to Area Under ROC Curve, F1-Score, and Accuracy

The area under the receiver operator characteristic curve (AUC) for each dataset and network-based machine learning model is shown in Table 2. Of the 17 datasets considered, the BNN, CNN, FNN, KAN, and SNN were the top-performing models in 4, 1, 5, 3, and 4 datasets, respectively. Table 3 displays the F1-score for each dataset and network-based machine learning model, where the BNN, CNN, FNN, KAN, and SNN were the top-performing models in 3, 3, 4, 4, and 3 datasets, respectively. The accuracy of each network-based machine learning model on each dataset is displayed in Table 4. For accuracy, the BNN, CNN, FNN, KAN, and SNN were the top-performing models in 4, 1, 4, 4, and 4 datasets, respectively. Note that Table 2, Table 3 and Table 4 report performance on both the train and test data. Receiver operator characteristic curves for each dataset and network-based model are shown in Supplementary Figure S1. Additionally, Supplementary File S2 contains tables depicting the precision, recall, sensitivity, and specificity of each network-based model on each dataset with respect to both train and test data.

### 3.2. Association of Top-Performing Network-Based Models with Dataset Characteristics

**Figure 3:**
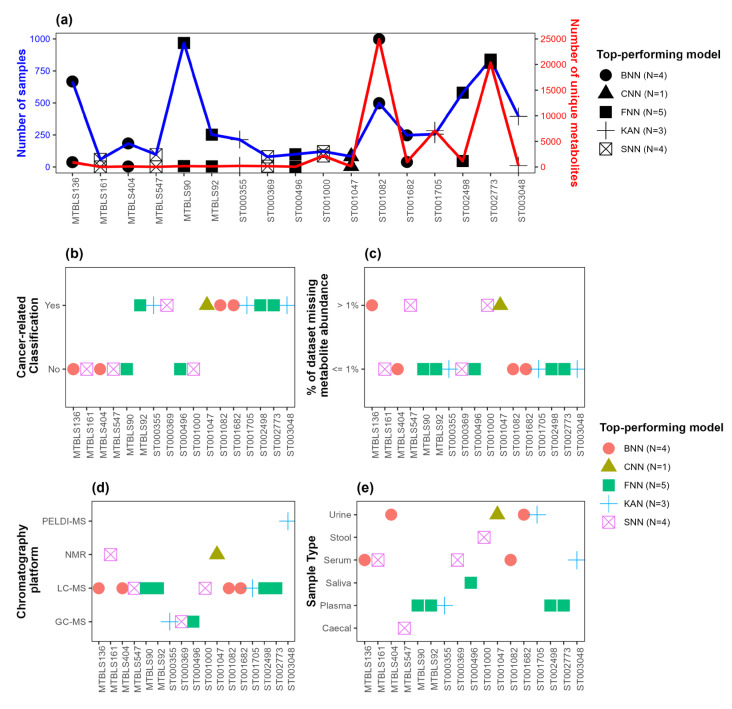
Top-performing models with respect to AUC. (**a**) Distribution of the number of observations (blue) and the number of predictor variables (red). (**b**) Distribution of an indicator variable indicating whether or not the binary classification for the given dataset is cancer-related. (**c**) Distribution of an indicator variable indicating whether the percentage of missing data is ≤1% or >1%. (**d**) Distribution of chromatography platforms. (**e**) Distribution of sample types. The shape and color of points indicate the top-performing network-based model. The number of datasets where each network-based model outperformed the others is indicated in the legend. BNN: Bayesian neural network; CNN: convolutional neural network; FNN: feedforward neural network; KAN: Kolmogorov–Arnold network; SNN: spiking neural network.

**Figure 4:**
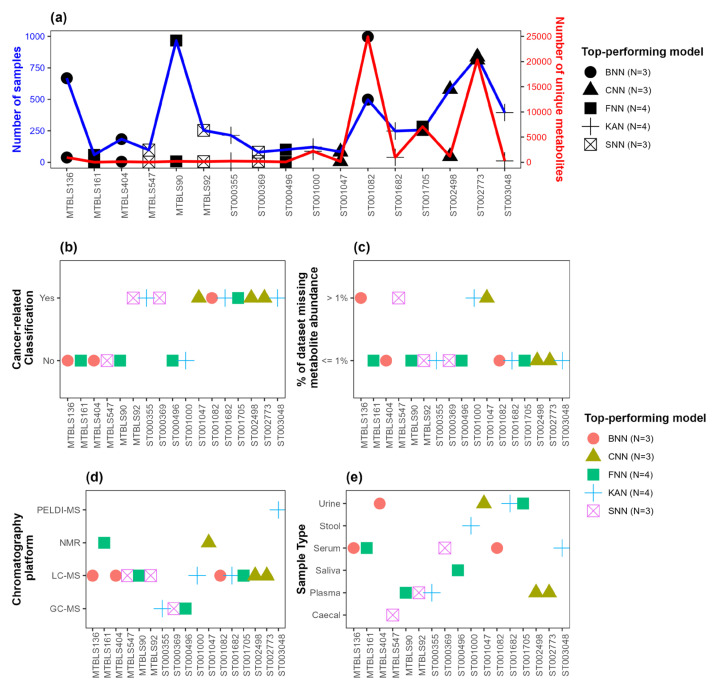
Top-performing models with respect to F1-score. (**a**) Distribution of the number of observations (blue) and the number of predictor variables (red). (**b**) Distribution of an indicator variable indicating whether the binary classification for the given dataset is cancer-related. (**c**) Distribution of an indicator variable indicating whether the percentage of missing data is ≤1% or >1%. (**d**) Distribution of chromatography platforms. (**e**) Distribution of sample types. The shape and color of points indicate the top-performing network-based model. The number of datasets where each network-based model outperformed the others is indicated in the legend. BNN: Bayesian neural network; CNN: convolutional neural network; FNN: feedforward neural network; KAN: Kolmogorov–Arnold network; SNN: spiking neural network.

**Figure 5:**
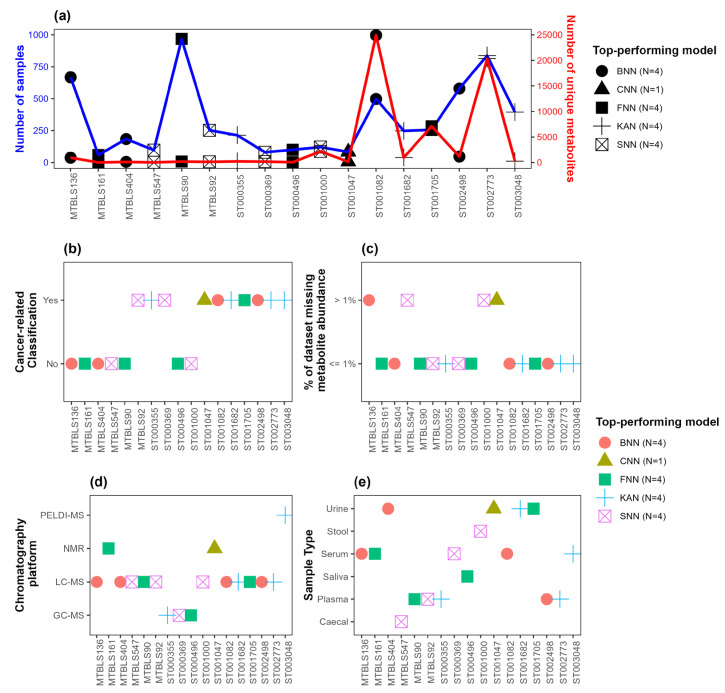
Top-performing models with respect to accuracy. (**a**) Distribution of the number of observations (blue) and the number of predictor variables (red). (**b**) Distribution of an indicator variable indicating whether the binary classification for the given dataset is cancer-related. (**c**) Distribution of an indicator variable indicating whether the percentage of missing data is ≤1% or >1%. (**d**) Distribution of chromatography platforms. (**e**) Distribution of sample types. The shape and color of points indicate the top-performing network-based model. The number of datasets where each network-based model outperformed the others is indicated in the legend. BNN: Bayesian neural network; CNN: convolutional neural network; FNN: feedforward neural network; KAN: Kolmogorov–Arnold network; SNN: spiking neural network.

The distribution of (i) the number of observations and (ii) the number of predictor variables (i.e., metabolites) in each dataset, along with the top-performing network-based model with respect to AUC, is depicted in Figure 3a. Figure 3b–e show the top-performing network-based models with respect to AUC along with dataset characteristics, including an indicator variable regarding whether the binary classification performed for the given dataset was cancer-related (Figure 3b), the percentage of missing metabolite abundance data(≤1% or >1%) (Figure 3c), the chromatography platform used (Figure 3d), and sample type (Figure 3e). Figure 4 and Figure 5 present similar information, showing the top-performing network-based models with respect to F1-score and accuracy, respectively. Note that all datasets where FNN was the top-performing model, regardless of whether AUC, F1-score, or accuracy was used as the evaluation metric, had minimal missing data (≤1%), as indicated in Figure 3c, Figure 4c and Figure 5c. Additionally, Figure 3b,c, Figure 4b,c and Figure 5b,c show that all datasets where KAN was the top-performing model involved cancer-related binary classification and had minimal missing data (≤1%), with a single exception when F1-score was considered as the evaluation metric. Lastly, note in Figure 3a, Figure 4a, and Figure 5a that SNN performed well in datasets with a relatively small number of observations and predictor variables.

The associations between top-performing models and dataset characteristics, including cancer flag, % NA, chromatography platform, sample type, number of samples, and number of metabolites, are summarized in Table 5. For categorical variables such as cancer flag (a binary variable indicating whether dataset included cancer-related groups), % NA (a binary variable indicating whether the dataset had ≤1% or >1% missing values), chromatography platform, and sample type, Fisher’s exact test was performed with respect to a binary variable, indicting whether each network-based model was the top-performing model for the given dataset and evaluation metric. To assess the association between the continuous variables (number of samples and number of metabolites) and the binary variable indicating top-performing network-based models, *t*-tests were performed. We observe several interesting associations. First, there is a significant association between the binary variable indicating whether CNN or SNN was the top-performing model and the continuous variable representing the number of samples when either AUC or accuracy is used as the evaluation metric. Additionally, there is a significant association between the binary variable indicating whether SNNs were the top-performing model and the number of metabolites when F1-score is used as the evaluation metric. There is also a significant association between the binary variable indicating whether FNNs were the top-performing model and the categorical variable representing sample type when AUC is used as the evaluation metric. Lastly, observe a significant association between number of metabolites and the binary variable indicating whether CNNs or SNNs were the top-performing models when accuracy or F1-score is used as the evaluation metric, respectively.

### 3.3. Computational Expense

A comparison of the computational expense of each network-based model for each dataset is given in Table 6. To quantify computational expense, one third of each dataset was randomly sampled without replacement 1000 times, and then each of the five network-based models with their optimal hyperparameters for the given dataset were evaluated on the randomly sampled subsets. The total time required to evaluate each model across the 1000 samplings is summarized in Table 6. We observe that BNN was the least computationally expensive network-based model in 15 of the 17 datasets.

## 4. Discussion

Of the 17 datasets considered in this study, BNNs, CNNs, FNNs, KANs, and SNNs were the top-performing models in 4, 3, 4; 1, 3, 1; 5, 4, 4; 3, 4, 4; and 4, 3, 4 datasets with respect to AUC, F1-score, and accuracy, respectively. The variability in top-performing models among the datasets makes the overlapping confidence intervals of AUC, F1-score, and accuracy on test datasets unsurprising. Additionally, network-based modeling approaches often perform better on datasets with many more observations than those used in this study. The relatively small sample sizes in some datasets may explain why the test AUC, F1-score, and accuracy were occasionally larger than the train AUC, F1-score, and accuracy, as observed in Table 3, Table 4 and Table 5, respectively.

Considering that BNNs were originally designed to improve uncertainty estimations in predictions and not necessarily prediction performance itself, it is perhaps unsurprising that BNNs were the top-performing network-based model in only 4, 3, and 4 datasets with respect to AUC, F1-score, and accuracy, respectively.

CNNs were the top-performing network-based model in only 1, 3, and 1 dataset(s) with respect to AUC, F1-score, and accuracy, respectively. CNNs are well-known for their strong performance in image classification, where each observation typically has dimension at least two. However, in metabolomics, data is inherently one-dimensional, with each sample containing quantified abundances of a set of metabolites. Unlike in images, where spatial relationships exist between a given pixel and the pixels in a neighborhood of that pixel, in metabolomics data, the data are inherently one-dimensional. Certain metabolites may be correlated but are not necessarily “close” to one another in the tabular data. This lack of spatial structure in metabolomics data may explain why CNNs did not outperform other network-based modeling approaches like they typically do in image classification.

FNNs outperformed all other network-based models in 5, 4, and 4 datasets with respect to AUC, F1-score, and accuracy, respectively. This suggests that while other network-based modeling approaches tend to outperform the simple FNN on various tasks such as quantifying prediction uncertainty (BNNs), working with higher-dimensional data (CNNs), function fitting (KANs), or imitating brain physiology/less energy consumption (SNNs), when working with tabular metabolomics data, FNNs can be a robust choice of network-based model to use.

KANs were the top-performing model in 3, 4, and 4 datasets with respect to AUC, F1-score, and accuracy, respectively. Given that KANs are primarily designed for function approximation rather than classification, perhaps it is not unexpected that KANs did not consistently outperform the other network-based modeling approaches in separating data into different categories.

FNNs and KANs performed better with datasets that had fewer missing data (<1%) when the same K-nearest neighbors imputation was applied across all datasets. This imputation approach was chosen for consistency with Mendez et al. [6], where the same approach was used for handling missing data. These results suggest that, for the 17 datasets considered, FNNs and KANs may be more sensitive to the quality and completeness of input data, making their generalizability more susceptible to missing data patterns compared to other network-based models. Additionally, when missing data are minimal, FNNs and KANs may better leverage reliable feature representations, leading to improved predictive performance on unseen test data.

SNNs, which more closely resemble brain physiology than other network-based models, were the top-performing model in only 4, 3, and 4 datasets with respect to AUC, F1-score, and accuracy, respectively. One advantage of SNNs is that given certain hardware (i.e., neuromorphic devices), they can be much more efficient than other network-based models. However, in applications where one may want to implement SNNs on such hardware, care must be taken to ensure that the performance of SNNs is not substantially worse than that of other machine learning modeling approaches.

## 5. Conclusions

Notably, no network-based model consistently outperformed the other network-based models, demonstrating that different network-based modeling approaches should be considered when designing network-based machine learning approaches to metabolomics-based classification. Futures directions may include applying other variations of neural networks such as modular neural networks or capsule neural networks to classification tasks using metabolomic data [83,84]. Given that network-based models tend to handle high-dimensional data well, integrating metabolomics data with other omics (or non-omics) data may hold utility. Overall, no network-based model in particular was determined to be the best approach to binary classification on metabolomics data, and much work is needed in order for network-based machine learning models to be more widely adopted in practice.

## Figures and Tables

**Table 1:** Dataset characteristics.

Dataset	Number of Samples (Cases/Controls)	Number of Metabolites	Abundance: [min, max]	Number of NA (%NA)	Species	Platform	Type	Comparison (Case/Control)
MTBLS136	668 (337/331)	949	[2065.0, 15,380,263,936.0]	126,047 (19.9%)	Humans	LC-MS	Serum	Postmenopausal hormone: estrogen/estrogen + progesterone
MTBLS161	59 (34/25)	29	[0.1, 1812.2]	0 (0.0%)	Humans	NMR	Serum	Chronic fatigue syndrome vs. not chronic fatigue syndrome
MTBLS404	184 (101/83)	120	[506.5, 678,587,872.0]	0 (0.0%)	Humans	LC-MS	Urine	Male vs. Female
MTBLS547	97 (46/51)	42	[0.0, 2.3]	105 (2.6%)	Mouse	LC-MS	Caecal	High fat diet vs. not high fat diet
MTBLS90	968 (485/483)	189	[4.0, 24.0]	0 (0.0%)	Humans	LC-MS	Plasma	Male vs. Female
MTBLS92	253 (111/142)	138	[0.0, 1856.5]	1 (0.0%)	Humans	LC-MS	Plasma	Before breast cancer therapy vs. after breast cancer therapy
ST000355	214 (138/76)	227	[0.0, 250.6]	0 (0.0%)	Humans	GC-MS	Plasma	Breast cancer vs. not breast cancer
ST000369	80 (49/31)	181	[6.0, 696,670.0]	8 (0.1%)	Humans	GC-MS	Serum	Adenocarcinoma vs. not adenocarcinoma
ST000496	100 (50/50)	69	[0.1, 1063.6]	0 (0.0%)	Humans	GC-MS	Saliva	Pre-debridement vs. post-debridement
ST001000	121 (68/53)	2177	[0.0, 236,557.0]	93,263 (35.4%)	Humans	LC-MS	Stool	Crohn’s disease vs. ulcerative colitis
ST001047	83 (43/40)	149	[0.1, 160,844.7]	696 (5.6%)	Humans	NMR	Urine	Gastric cancer vs. not gastric cancer
ST001082	498 (228/270)	24,928	[0.0, 236,619,108.7]	498 (0.0%)	Humans	LC-MS	Serum	Prostate cancer recurrence vs. not recurrence
ST001682	248 (128/120)	982	[1803.4, 64,128,929.1]	0 (0.0%)	Humans	LC-MS	Urine	Bladder cancer vs. not bladder cancer
ST001705	256 (82/174)	7097	[20.0, 6,474,764,723.0]	0 (0.0%)	Humans	LC-MS	Urine	Renal cell carcinoma vs. not renal cell carcinoma
ST002498	580 (267/313)	1169	[12.0, 32.6]	0 (0.0%)	Humans	LC-MS	Plasma	Prostate cancer vs. not prostate cancer
ST002773	838 (410/428)	20,342	[0.0, 7,094,237,770.0]	0 (0.0%)	Humans	LC-MS	Plasma	Lung cancer vs. not lung cancer in never-smoking women
ST003048	395 (191/204)	272	[−11,995.1, 373,965.3]	0 (0.0%)	Humans	PELDI-MS	Serum	Endometrial cancer vs. not endometrial cancer

**Table 2:** AUC and 95% confidence intervals (CIs) across network-based models and datasets. CIs for the training data were computed from the AUCs of the 50 folds (derived from 5-fold stratified cross-validation with 10 different partitions). CIs for the test data were calculated using the bootstrap method with 10,000 resamplings. For each dataset, the largest AUC on the test data across the network-based models is highlighted in bold. BNN: Bayesian neural network; CNN: convolutional neural network; FNN: feedforward neural network; KAN: Kolmogorov–Arnold network; SNN: spiking neural network.

Dataset		BNN	CNN	FNN	KAN	SNN
MTBLS136	Train	0.786 [0.714,0.862]	0.741 [0.666,0.818]	0.782 [0.712,0.853]	0.741 [0.636,0.828]	0.758 [0.668,0.833]
	Test	**0.792 [0.734,0.850]**	0.757 [0.694,0.819]	0.760 [0.698,0.822]	0.747 [0.682,0.812]	0.707 [0.640,0.774]
MTBLS161	Train	0.819 [0.500,1.000]	0.842 [0.522,1.000]	0.827 [0.522,1.000]	0.850 [0.606,1.000]	0.821 [0.587,1.000]
	Test	0.917 [0.789,1.000]	0.844 [0.633,1.000]	0.875 [0.698,1.000]	0.500 [0.262,0.738]	**0.932 [0.815,1.000]**
MTBLS404	Train	0.944 [0.874,1.000]	0.870 [0.736,0.986]	0.942 [0.867,1.000]	0.912 [0.805,1.000]	0.921 [0.818,1.000]
	Test	**0.947 [0.900,0.995]**	0.853 [0.754,0.952]	0.919 [0.856,0.982]	0.827 [0.722,0.932]	0.901 [0.826,0.975]
MTBLS547	Train	0.945 [0.833,1.000]	0.947 [0.833,1.000]	0.942 [0.787,1.000]	0.919 [0.753,1.000]	0.945 [0.812,1.000]
	Test	0.886 [0.758,1.000]	0.860 [0.726,0.995]	0.864 [0.729,0.999]	0.842 [0.697,0.987]	**0.925 [0.842,1.000]**
MTBLS90	Train	0.824 [0.776,0.875]	0.771 [0.684,0.827]	0.827 [0.773,0.876]	0.792 [0.683,0.871]	0.805 [0.738,0.867]
	Test	0.833 [0.790,0.877]	0.845 [0.802,0.887]	**0.860 [0.818,0.902]**	0.551 [0.488,0.614]	0.811 [0.765,0.857]
MTBLS92	Train	0.846 [0.730,0.943]	0.787 [0.674,0.898]	0.844 [0.738,0.931]	0.833 [0.741,0.939]	0.819 [0.675,0.940]
	Test	0.773 [0.671,0.874]	0.694 [0.580,0.807]	**0.788 [0.690,0.885]**	0.661 [0.547,0.775]	0.748 [0.643,0.853]
ST000355	Train	0.984 [0.906,1.000]	0.963 [0.895,1.000]	0.983 [0.905,1.000]	0.982 [0.914,1.000]	0.986 [0.919,1.000]
	Test	0.980 [0.957,1.000]	0.938 [0.871,1.000]	0.984 [0.964,1.000]	**0.986 [0.964,1.000]**	0.981 [0.957,1.000]
ST000369	Train	0.748 [0.465,0.992]	0.755 [0.536,0.932]	0.740 [0.534,0.991]	0.732 [0.464,0.926]	0.715 [0.523,0.942]
	Test	0.812 [0.622,1.000]	0.876 [0.732,1.000]	0.871 [0.726,1.000]	0.582 [0.302,0.863]	**0.924 [0.823,1.000]**
ST000496	Train	0.951 [0.773,1.000]	0.917 [0.762,1.000]	0.945 [0.810,1.000]	0.860 [0.712,1.000]	0.923 [0.791,1.000]
	Test	0.958 [0.901,1.000]	0.782 [0.625,0.939]	**0.965 [0.910,1.000]**	0.955 [0.879,1.000]	0.943 [0.872,1.000]
ST001000	Train	0.808 [0.623,0.968]	0.830 [0.667,0.968]	0.804 [0.591,0.977]	0.777 [0.543,0.968]	0.795 [0.592,0.945]
	Test	0.682 [0.514,0.851]	0.674 [0.507,0.840]	0.714 [0.554,0.873]	0.649 [0.476,0.821]	**0.754 [0.606,0.901]**
ST001047	Train	0.948 [0.808,1.000]	0.892 [0.643,1.000]	0.937 [0.808,1.000]	0.904 [0.537,1.000]	0.923 [0.689,1.000]
	Test	0.856 [0.716,0.997]	**0.949 [0.868,1.000]**	0.836 [0.687,0.984]	0.851 [0.710,0.992]	0.813 [0.655,0.971]
ST001082	Train	0.995 [0.970,1.000]	0.992 [0.936,1.000]	0.994 [0.967,1.000]	0.553 [0.398,0.711]	0.968 [0.914,0.997]
	Test	**0.999 [0.998,1.000]**	0.997 [0.993,1.000]	0.999 [0.997,1.000]	0.580 [0.498,0.661]	0.991 [0.979,1.000]
ST001682	Train	0.597 [0.489,0.745]	0.599 [0.477,0.768]	0.596 [0.476,0.756]	0.603 [0.501,0.744]	0.597 [0.442,0.774]
	Test	**0.637 [0.511,0.764]**	0.609 [0.484,0.734]	0.573 [0.444,0.702]	0.603 [0.479,0.727]	0.615 [0.504,0.726]
ST001705	Train	0.974 [0.924,1.000]	0.982 [0.950,1.000]	0.977 [0.928,1.000]	0.980 [0.916,1.000]	0.966 [0.893,1.000]
	Test	0.982 [0.960,1.000]	0.978 [0.944,1.000]	0.982 [0.953,1.000]	**0.991 [0.979,1.000]**	0.953 [0.912,0.993]
ST002498	Train	0.561 [0.489,0.641]	0.558 [0.472,0.650]	0.554 [0.470,0.648]	0.560 [0.495,0.643]	0.560 [0.493,0.674]
	Test	0.558 [0.476,0.640]	0.507 [0.424,0.590]	**0.561 [0.480,0.641]**	0.556 [0.478,0.633]	0.547 [0.469,0.625]
ST002773	Train	0.582 [0.491,0.666]	0.602 [0.483,0.704]	0.594 [0.478,0.677]	0.540 [0.540,0.540]	0.568 [0.449,0.649]
	Test	0.591 [0.524,0.658]	0.604 [0.537,0.671]	**0.611 [0.546,0.677]**	0.523 [0.456,0.591]	0.577 [0.511,0.644]
ST003048	Train	0.966 [0.920,0.996]	0.933 [0.871,0.978]	0.967 [0.910,0.994]	0.939 [0.869,0.992]	0.948 [0.883,0.988]
	Test	0.940 [0.899,0.981]	0.895 [0.838,0.952]	0.951 [0.912,0.989]	**0.969 [0.941,0.997]**	0.963 [0.937,0.988]

**Table 3:** F1-score and 95% CIs across network-based models and datasets. CIs for the training data were computed from the F1-scores of the 50 folds (derived from 5-fold stratified cross-validation with 10 different partitions). CIs for the test data were calculated using the bootstrap method with 10,000 resamplings. For each dataset, the highest F1-score on the test data across the network-based models is highlighted in bold. BNN: Bayesian neural network; CNN: convolutional neural network; FNN: feedforward neural network; KAN: Kolmogorov–Arnold network; SNN: spiking neural network.

Dataset		BNN	CNN	FNN	KAN	SNN
MTBLS136	Train	0.717 [0.638,0.804]	0.671 [0.541,0.761]	0.712 [0.611,0.806]	0.680 [0.612,0.781]	0.690 [0.602,0.764]
	Test	**0.698 [0.622,0.765]**	0.670 [0.587,0.739]	0.673 [0.597,0.736]	0.697 [0.624,0.760]	0.635 [0.552,0.709]
MTBLS161	Train	0.708 [0.348,1.000]	0.700 [0.347,1.000]	0.742 [0.423,1.000]	0.738 [0.516,1.000]	0.679 [0.333,0.857]
	Test	0.769 [0.429,1.000]	0.750 [0.427,0.941]	**0.857 [0.571,1.000]**	0.333 [0.154,0.667]	0.800 [0.500,1.000]
MTBLS404	Train	0.851 [0.706,0.957]	0.756 [0.563,0.907]	0.859 [0.700,1.000]	0.846 [0.714,1.000]	0.821 [0.667,0.956]
	Test	**0.814 [0.682,0.906]**	0.733 [0.596,0.847]	0.787 [0.643,0.889]	0.724 [0.581,0.839]	0.759 [0.612,0.866]
MTBLS547	Train	0.899 [0.732,1.000]	0.900 [0.690,1.000]	0.896 [0.737,1.000]	0.855 [0.615,1.000]	0.887 [0.727,1.000]
	Test	**0.812 [0.625,0.947]**	**0.812 [0.643,0.945]**	0.788 [0.583,0.919]	0.788 [0.609,0.909]	**0.812 [0.621,0.941]**
MTBLS90	Train	0.752 [0.692,0.807]	0.713 [0.637,0.772]	0.775 [0.702,0.826]	0.709 [0.427,0.800]	0.751 [0.702,0.811]
	Test	0.702 [0.636,0.760]	0.784 [0.728,0.832]	**0.806 [0.759,0.850]**	0.665 [0.620,0.713]	0.747 [0.686,0.799]
MTBLS92	Train	0.796 [0.688,0.892]	0.766 [0.607,0.868]	0.804 [0.688,0.900]	0.809 [0.689,0.882]	0.780 [0.667,0.900]
	Test	0.717 [0.608,0.814]	0.673 [0.559,0.769]	**0.725 [0.615,0.813]**	0.688 [0.578,0.784]	**0.725 [0.608,0.816]**
ST000355	Train	0.950 [0.857,1.000]	0.898 [0.756,1.000]	0.959 [0.874,1.000]	0.930 [0.767,1.000]	0.950 [0.889,1.000]
	Test	0.909 [0.818,0.980]	0.840 [0.696,0.945]	0.863 [0.744,0.951]	**0.962 [0.902,1.000]**	0.943 [0.867,1.000]
ST000369	Train	0.594 [0.286,0.889]	0.533 [0.226,0.750]	0.590 [0.333,0.800]	0.550 [0.228,0.889]	0.537 [0.250,0.883]
	Test	0.640 [0.375,0.839]	**0.818 [0.583,0.960]**	0.727 [0.462,0.923]	0.476 [0.153,0.706]	**0.818 [0.625,0.960]**
ST000496	Train	0.887 [0.714,1.000]	0.829 [0.708,0.985]	0.888 [0.727,1.000]	0.829 [0.627,0.933]	0.857 [0.677,1.000]
	Test	**0.914 [0.800,1.000]**	0.743 [0.538,0.889]	**0.914 [0.800,1.000]**	0.865 [0.733,0.963]	0.848 [0.690,0.963]
ST001000	Train	0.646 [0.320,0.833]	0.637 [0.340,0.908]	0.674 [0.470,0.908]	0.658 [0.400,0.833]	0.671 [0.340,0.831]
	Test	0.438 [0.207,0.632]	0.483 [0.231,0.690]	0.500 [0.250,0.700]	**0.650 [0.444,0.800]**	0.588 [0.357,0.765]
ST001047	Train	0.861 [0.615,1.000]	0.766 [0.500,0.923]	0.843 [0.600,1.000]	0.808 [0.334,1.000]	0.835 [0.545,1.000]
	Test	0.800 [0.593,0.938]	**0.923 [0.778,1.000]**	0.741 [0.500,0.909]	0.741 [0.522,0.900]	0.759 [0.526,0.909]
ST001082	Train	0.970 [0.902,1.000]	0.969 [0.926,1.000]	0.971 [0.909,1.000]	0.642 [0.202,0.722]	0.904 [0.813,0.973]
	Test	**0.983 [0.960,1.000]**	0.978 [0.951,0.995]	0.977 [0.953,0.995]	0.545 [0.451,0.627]	0.955 [0.922,0.982]
ST001682	Train	0.403 [0.214,0.588]	0.408 [0.248,0.575]	0.402 [0.163,0.616]	0.411 [0.154,0.624]	0.405 [0.242,0.604]
	Test	0.346 [0.206,0.465]	0.424 [0.282,0.547]	0.410 [0.261,0.544]	**0.558 [0.427,0.674]**	0.348 [0.197,0.486]
ST001705	Train	0.935 [0.837,1.000]	0.943 [0.872,1.000]	0.951 [0.895,1.000]	0.958 [0.900,1.000]	0.929 [0.820,0.995]
	Test	0.966 [0.928,0.992]	0.951 [0.909,0.984]	**0.974 [0.938,1.000]**	0.966 [0.926,0.992]	0.870 [0.792,0.933]
ST002498	Train	0.531 [0.414,0.642]	0.561 [0.453,0.686]	0.549 [0.379,0.663]	0.582 [0.445,0.673]	0.534 [0.403,0.657]
	Test	0.592 [0.510,0.667]	**0.643 [0.568,0.704]**	0.588 [0.509,0.664]	0.621 [0.549,0.691]	0.569 [0.485,0.646]
ST002773	Train	0.462 [0.343,0.557]	0.444 [0.215,0.629]	0.472 [0.369,0.564]	0.676 [0.675,0.679]	0.444 [0.337,0.534]
	Test	0.390 [0.316,0.464]	**0.503 [0.436,0.572]**	0.409 [0.330,0.482]	0.419 [0.333,0.498]	0.498 [0.431,0.563]
ST003048	Train	0.912 [0.822,0.964]	0.878 [0.802,0.945]	0.923 [0.838,0.964]	0.896 [0.825,0.958]	0.905 [0.834,0.958]
	Test	0.899 [0.840,0.946]	0.863 [0.789,0.916]	0.902 [0.844,0.951]	**0.926 [0.881,0.969]**	0.877 [0.807,0.930]

**Table 4:** Accuracy and 95% CIs across network-based models and datasets. CIs for the training data were computed from the accuracies of the 50 folds (derived from 5-fold stratified cross-validation with 10 different partitions). CIs for the test data were calculated using the bootstrap method with 10,000 resamplings. For each dataset, the highest accuracy on the test data across the network-based models is highlighted in bold. BNN: Bayesian neural network; CNN: convolutional neural network; FNN: feedforward neural network; KAN: Kolmogorov–Arnold network; SNN: spiking neural network.

Dataset		BNN	CNN	FNN	KAN	SNN
MTBLS136	Train	0.718 [0.634,0.804]	0.676 [0.607,0.762]	0.713 [0.632,0.795]	0.683 [0.607,0.784]	0.703 [0.629,0.773]
	Test	**0.713 [0.650,0.771]**	0.691 [0.628,0.749]	0.677 [0.614,0.735]	0.704 [0.646,0.767]	0.655 [0.587,0.717]
MTBLS161	Train	0.727 [0.500,1.000]	0.739 [0.500,1.000]	0.756 [0.445,1.000]	0.763 [0.571,1.000]	0.708 [0.429,0.875]
	Test	0.850 [0.650,1.000]	0.800 [0.600,0.950]	**0.900 [0.750,1.000]**	0.600 [0.400,0.800]	0.850 [0.700,1.000]
MTBLS404	Train	0.871 [0.792,0.960]	0.786 [0.667,0.917]	0.879 [0.750,1.000]	0.866 [0.760,1.000]	0.842 [0.718,0.960]
	Test	**0.823 [0.726,0.903]**	0.742 [0.629,0.855]	0.790 [0.677,0.887]	0.742 [0.629,0.839]	0.774 [0.661,0.871]
MTBLS547	Train	0.897 [0.710,1.000]	0.897 [0.710,1.000]	0.896 [0.769,1.000]	0.855 [0.692,1.000]	0.888 [0.769,1.000]
	Test	**0.818 [0.667,0.939]**	**0.818 [0.696,0.939]**	0.788 [0.636,0.909]	0.788 [0.636,0.909]	**0.818 [0.667,0.939]**
MTBLS90	Train	0.754 [0.690,0.814]	0.711 [0.627,0.758]	0.775 [0.709,0.820]	0.713 [0.392,0.804]	0.755 [0.699,0.818]
	Test	0.737 [0.690,0.783]	0.780 [0.734,0.824]	**0.808 [0.768,0.851]**	0.498 [0.449,0.554]	0.765 [0.718,0.808]
MTBLS92	Train	0.769 [0.654,0.876]	0.735 [0.565,0.843]	0.773 [0.624,0.882]	0.787 [0.667,0.853]	0.752 [0.624,0.879]
	Test	0.694 [0.600,0.800]	0.624 [0.518,0.718]	**0.706 [0.612,0.800]**	0.647 [0.541,0.753]	**0.706 [0.600,0.800]**
ST000355	Train	0.963 [0.893,1.000]	0.933 [0.857,1.000]	0.972 [0.901,1.000]	0.954 [0.869,1.000]	0.966 [0.929,1.000]
	Test	0.931 [0.861,0.986]	0.889 [0.806,0.958]	0.903 [0.833,0.958]	**0.972 [0.931,1.000]**	0.958 [0.903,1.000]
ST000369	Train	0.648 [0.465,0.909]	0.649 [0.323,0.818]	0.622 [0.400,0.818]	0.667 [0.384,0.909]	0.631 [0.455,0.909]
	Test	0.667 [0.481,0.852]	**0.852 [0.704,0.963]**	0.778 [0.630,0.926]	0.593 [0.407,0.778]	**0.852 [0.704,0.963]**
ST000496	Train	0.888 [0.710,1.000]	0.822 [0.633,0.984]	0.888 [0.769,1.000]	0.824 [0.622,0.923]	0.856 [0.692,1.000]
	Test	**0.912 [0.824,1.000]**	0.735 [0.588,0.882]	**0.912 [0.824,1.000]**	0.853 [0.735,0.971]	0.853 [0.735,0.971]
ST001000	Train	0.706 [0.514,0.875]	0.709 [0.514,0.923]	0.715 [0.562,0.923]	0.705 [0.562,0.875]	0.716 [0.514,0.861]
	Test	0.561 [0.390,0.707]	0.634 [0.488,0.780]	0.610 [0.463,0.756]	**0.659 [0.488,0.805]**	**0.659 [0.512,0.805]**
ST001047	Train	0.864 [0.636,1.000]	0.776 [0.566,0.909]	0.847 [0.636,1.000]	0.827 [0.545,1.000]	0.845 [0.545,1.000]
	Test	0.786 [0.607,0.929]	**0.929 [0.821,1.000]**	0.750 [0.571,0.893]	0.750 [0.571,0.893]	0.750 [0.571,0.894]
ST001082	Train	0.967 [0.894,1.000]	0.966 [0.924,1.000]	0.969 [0.901,1.000]	0.532 [0.392,0.637]	0.900 [0.807,0.970]
	Test	**0.982 [0.958,1.000]**	0.976 [0.952,0.994]	0.976 [0.952,0.994]	0.518 [0.446,0.596]	0.952 [0.922,0.982]
ST001682	Train	0.438 [0.280,0.576]	0.436 [0.303,0.569]	0.442 [0.303,0.569]	0.448 [0.280,0.606]	0.442 [0.280,0.599]
	Test	0.361 [0.253,0.482]	0.410 [0.301,0.506]	0.446 [0.337,0.554]	**0.542 [0.434,0.651]**	0.458 [0.361,0.566]
ST001705	Train	0.914 [0.801,1.000]	0.918 [0.801,1.000]	0.933 [0.853,1.000]	0.942 [0.853,1.000]	0.909 [0.778,0.993]
	Test	0.953 [0.907,0.988]	0.930 [0.872,0.977]	**0.965 [0.919,1.000]**	0.953 [0.907,0.988]	0.837 [0.756,0.907]
ST002498	Train	0.507 [0.394,0.623]	0.507 [0.406,0.597]	0.512 [0.393,0.611]	0.537 [0.419,0.633]	0.502 [0.381,0.619]
	Test	**0.552 [0.485,0.624]**	0.541 [0.474,0.608]	0.531 [0.459,0.608]	0.541 [0.474,0.608]	0.531 [0.464,0.603]
ST002773	Train	0.450 [0.369,0.530]	0.432 [0.361,0.508]	0.438 [0.348,0.523]	0.511 [0.509,0.514]	0.435 [0.367,0.495]
	Test	0.407 [0.350,0.464]	0.450 [0.396,0.511]	0.411 [0.354,0.468]	**0.525 [0.468,0.586]**	0.432 [0.379,0.489]
ST003048	Train	0.910 [0.820,0.962]	0.873 [0.797,0.943]	0.921 [0.838,0.962]	0.893 [0.828,0.958]	0.904 [0.834,0.958]
	Test	0.894 [0.841,0.939]	0.856 [0.788,0.909]	0.902 [0.848,0.947]	**0.924 [0.879,0.970]**	0.879 [0.818,0.932]

**Table 5:** Association between top-performing models and dataset characteristics. Values represent *p*-values from Fisher’s exact tests for categorical variables (cancer flag, % NA, platform, and sample type) and *t*-tests for continuous variables (number of samples and number of metabolites). For F1-score and accuracy, the natural logarithm transformation was applied to the number of samples and metabolites before conducting the *t*-tests. *p*-values less than 0.05 are highlighted in bold. BNN: Bayesian neural network; CNN: convolutional neural network; FNN: feedforward neural network; KAN: Kolmogorov–Arnold network; SNN: spiking neural network.

Dataset Characteristic	Evaluation Metric	BNN	CNN	FNN	KAN	SNN
Cancer Flag	AUC	1.000	1.000	1.000	0.228	0.250
	F1-score	0.537	0.228	0.250	0.603	1.000
	Accuracy	1.000	1.000	0.250	0.103	1.000
% NA	AUC	1.000	0.235	0.261	0.541	0.219
	F1-score	1.000	1.000	0.519	1.000	1.000
	Accuracy	1.000	0.235	0.519	0.519	0.219
Platform	AUC	0.792	0.176	1.000	0.175	0.792
	F1-score	1.000	0.515	0.792	0.376	1.000
	Accuracy	0.792	0.176	0.792	0.376	1.000
Sample Type	AUC	0.601	0.412	**0.009**	1.000	0.074
	F1-score	0.706	0.706	0.769	0.769	0.500
	Accuracy	1.000	0.412	0.769	1.000	0.172
Number of Samples	AUC	0.549	**0.002**	0.141	0.604	**0.002**
	F1-score	0.242	0.587	0.742	0.893	0.151
	Accuracy	0.067	**<0.001**	0.742	0.186	**0.044**
Number of Metabolites	AUC	0.538	0.084	0.784	0.728	0.139
	F1-score	0.492	0.433	0.500	0.725	**0.026**
	Accuracy	0.324	**0.030**	0.500	0.424	0.350

**Table 6:** Computational time for network-based models on random subsets. Each dataset was randomly sampled without replacement into one-third subsets 1000 times. Each of the five network-based models with their optimal hyperparameters for the given dataset were evaluated on these subsets. The time required to evaluate the models on each subset was recorded in minutes. Mean times with 95% CIs are reported. For each dataset, the least computational expense is highlighted in bold. BNN: Bayesian neural network; CNN: convolutional neural network; FNN: feedforward neural network; KAN: Kolmogorov–Arnold Network; SNN: spiking neural network.

Dataset	BNN	CNN	FNN	KAN	SNN
MTBLS136	0.08 [0.02,0.19]	0.25 [0.13,0.32]	**0.05 [0.05,0.06]**	2.02 [1.46,2.72]	3.14 [3.09,3.19]
MTBLS161	**0.01 [0.01,0.01]**	0.08 [0.05,0.12]	0.08 [0.08,0.08]	0.01 [0.01,0.02]	0.90 [0.88,0.92]
MTBLS404	**0.01 [0.01,0.01]**	0.17 [0.08,0.23]	0.04 [0.04,0.04]	0.18 [0.17,0.20]	1.48 [1.46,1.51]
MTBLS547	**0.01 [0.01,0.01]**	0.14 [0.09,0.21]	0.08 [0.08,0.08]	0.02 [0.02,0.03]	0.81 [0.80,0.83]
MTBLS90	**0.00 [0.00,0.01]**	0.17 [0.12,0.23]	0.08 [0.06,0.11]	0.20 [0.18,0.23]	4.18 [4.12,4.25]
MTBLS92	**0.00 [0.00,0.00]**	0.25 [0.22,0.29]	0.08 [0.08,0.08]	0.16 [0.15,0.19]	1.17 [1.15,1.19]
ST000355	**0.01 [0.01,0.01]**	0.26 [0.22,0.32]	0.08 [0.08,0.08]	0.08 [0.08,0.10]	0.99 [0.97,1.00]
ST000369	**0.00 [0.00,0.00]**	0.14 [0.08,0.21]	0.04 [0.04,0.04]	0.07 [0.06,0.08]	0.61 [0.60,0.62]
ST000496	**0.01 [0.01,0.01]**	0.17 [0.09,0.23]	0.08 [0.08,0.08]	0.01 [0.01,0.02]	0.42 [0.41,0.43]
ST001000	**0.01 [0.01,0.01]**	0.13 [0.11,0.15]	0.08 [0.08,0.08]	0.25 [0.24,0.29]	0.97 [0.96,0.99]
ST001047	**0.01 [0.01,0.01]**	0.14 [0.08,0.21]	0.04 [0.04,0.04]	0.02 [0.01,0.02]	1.27 [1.25,1.31]
ST001082	0.14 [0.13,0.16]	16.30 [13.52,17.08]	**0.13 [0.11,0.20]**	11.19 [6.76,25.14]	24.25 [22.92,24.90]
ST001682	**0.01 [0.01,0.01]**	0.27 [0.22,0.33]	0.05 [0.05,0.05]	0.34 [0.23,0.44]	2.11 [2.08,2.14]
ST001705	**0.07 [0.06,0.07]**	0.92 [0.61,1.05]	0.08 [0.08,0.11]	2.90 [1.85,4.83]	2.80 [2.73,2.86]
ST002498	**0.01 [0.01,0.02]**	0.40 [0.26,0.51]	0.05 [0.05,0.08]	1.81 [0.81,11.83]	5.68 [5.58,5.83]
ST002773	**0.15 [0.14,0.17]**	18.26 [13.31,22.10]	0.22 [0.22,0.23]	18.22 [12.52,25.47]	28.82 [22.62,63.62]
ST003048	**0.01 [0.01,0.01]**	0.27 [0.22,0.34]	0.04 [0.04,0.05]	2.47 [0.30,12.40]	5.94 [3.01,50.26]

## Data Availability

All datasets used in this study are free and publicly available. Of the 17 datasets used in this study, 11 can be found in “Metabolomics Workbench”, and 6 can be found in “MetaboLights” [63,85]. All code used in this project is free and publicly available and can be found on GitHub: https://github.com/hdlugas/NNs_for_binary_classification.

## Supplementary Materials

The following supporting information can be downloaded at: https://www.mdpi.com/article/10.3390/metabo15030174/s1, Figure S1: ROC curves from test data with AUC and 95% confidence intervals computed from bootstrap method with 1000 resamplings. File S2: Tables displaying precision, recall, sensitivity, and specificity of each network-based machine learning model for each dataset.
