# Supplementary material for: A Comparative Study of Network-Based Machine Learning Approaches for Binary Classification in Metabolomics
Source: Metabolites. 2025 Mar 3;15(3):174. doi: 10.3390/metabo15030174 (PMC11944042; doi:10.3390/metabo15030174)
Supplement: Supplementary file 1 [file metabolites-15-00174-s001.zip › supplementary/S2.pdf]

S2: Supplementary File 2

Table S1. Precision and 95% confidence intervals (CIs) across network-based models and datasets. CIs for the training data were computed from the precisions of the 50 folds (derived from 5-fold stratified cross-validation with 10 different partitions). CIs for the test data were calculated using the bootstrap method with 10,000 resamplings. For each dataset, the largest precision on the test data across the network-based models is highlighted in bold. BNN: Bayesian Neural Network; CNN: Convolutional Neural Network; FNN: Feedforward Neural Network; KAN: Kolmogorov-Arnold Network; SNN: Spiking Neural Network.

Dataset		BNN	CNN	FNN	KAN	SNN
MTBLS136	Train	0.716 [0.716,0.716]	0.678 [0.678,0.678]	0.711 [0.711,0.711]	0.684 [0.684,0.684]	0.719 [0.719,0.719]
	Test	**0.725 [0.638,0.810]**	0.707 [0.613,0.792]	0.673 [0.586,0.758]	0.704 [0.618,0.789]	0.663 [0.562,0.750]
MTBLS161	Train	0.698 [0.698,0.698]	0.741 [0.741,0.741]	0.739 [0.739,0.739]	0.758 [0.758,0.758]	0.682 [0.682,0.682]
	Test	**1.000 [1.000,1.000]**	0.750 [0.400,1.000]	**1.000 [1.000,1.000]**	0.500 [0.000,1.000]	0.857 [0.500,1.000]
MTBLS404	Train	0.882 [0.882,0.882]	0.783 [0.783,0.783]	0.889 [0.889,0.889]	0.876 [0.876,0.876]	0.833 [0.833,0.833]
	Test	**0.774 [0.613,0.900]**	0.688 [0.515,0.846]	0.727 [0.562,0.875]	0.700 [0.536,0.871]	0.733 [0.567,0.885]
MTBLS547	Train	0.938 [0.938,0.938]	0.916 [0.916,0.916]	0.931 [0.931,0.931]	0.903 [0.903,0.903]	0.935 [0.935,0.935]
	Test	**0.867 [0.667,1.000]**	**0.867 [0.667,1.000]**	0.812 [0.615,1.000]	0.812 [0.600,1.000]	**0.867 [0.692,1.000]**
MTBLS90	Train	0.760 [0.760,0.760]	0.710 [0.710,0.710]	0.776 [0.776,0.776]	0.727 [0.727,0.727]	0.763 [0.763,0.763]
	Test	0.806 [0.733,0.873]	0.768 [0.702,0.830]	**0.811 [0.752,0.868]**	0.498 [0.446,0.551]	0.806 [0.736,0.871]
MTBLS92	Train	0.785 [0.785,0.785]	0.759 [0.759,0.759]	0.778 [0.778,0.778]	0.812 [0.812,0.812]	0.775 [0.775,0.775]
	Test	0.750 [0.619,0.884]	0.660 [0.523,0.800]	**0.767 [0.643,0.881]**	0.688 [0.565,0.826]	**0.767 [0.625,0.891]**
ST000355	Train	0.936 [0.936,0.936]	0.949 [0.949,0.949]	0.977 [0.977,0.977]	0.968 [0.968,0.968]	0.964 [0.964,0.964]

Dataset		BNN	CNN	FNN	KAN	SNN
	Test	0.862<br>[0.719,0.969]	0.875<br>[0.722,1.000]	0.880<br>[0.727,1.000]	**0.962**<br>**[0.871,1.000]**	0.926<br>[0.815,1.000]
ST000369	Train	0.544<br>[0.544,0.544]	0.589<br>[0.589,0.589]	0.555<br>[0.555,0.555]	0.622<br>[0.622,0.622]	0.565<br>[0.565,0.565]
	Test	0.533<br>[0.294,0.786]	**0.750**<br>**[0.499,1.000]**	0.667<br>[0.400,0.917]	0.455<br>[0.154,0.750]	**0.750**<br>**[0.500,1.000]**
ST000496	Train	0.881<br>[0.881,0.881]	0.815<br>[0.815,0.815]	0.885<br>[0.885,0.885]	0.814<br>[0.814,0.814]	0.853<br>[0.853,0.853]
	Test	**0.889**<br>**[0.714,1.000]**	0.722<br>[0.500,0.929]	**0.889**<br>**[0.733,1.000]**	0.800<br>[0.619,0.950]	0.875<br>[0.684,1.000]
ST001000	Train	0.690<br>[0.690,0.690]	0.702<br>[0.702,0.702]	0.692<br>[0.692,0.692]	0.683<br>[0.683,0.683]	0.685<br>[0.685,0.685]
	Test	0.500<br>[0.231,0.765]	**0.636**<br>**[0.333,0.923]**	0.571<br>[0.273,0.833]	0.591<br>[0.381,0.792]	0.625<br>[0.385,0.857]
ST001047	Train	0.854<br>[0.854,0.854]	0.780<br>[0.780,0.780]	0.836<br>[0.836,0.836]	0.835<br>[0.835,0.835]	0.860<br>[0.860,0.860]
	Test	0.706<br>[0.462,0.929]	**0.923**<br>**[0.750,1.000]**	0.714<br>[0.462,0.933]	0.714<br>[0.455,0.923]	0.688<br>[0.499,0.889]
ST001082	Train	0.972<br>[0.972,0.972]	0.968<br>[0.968,0.968]	0.970<br>[0.970,0.970]	0.557<br>[0.557,0.557]	0.940<br>[0.940,0.940]
	Test	0.989<br>[0.959,1.000]	0.978<br>[0.944,1.000]	**1.000**<br>**[1.000,1.000]**	0.558<br>[0.456,0.667]	0.977<br>[0.942,1.000]
ST001682	Train	0.412<br>[0.412,0.412]	0.415<br>[0.415,0.415]	0.418<br>[0.418,0.418]	0.428<br>[0.428,0.428]	0.422<br>[0.422,0.422]
	Test	0.341<br>[0.194,0.487]	0.400<br>[0.261,0.537]	0.421<br>[0.265,0.579]	**0.522**<br>**[0.381,0.673]**	0.414<br>[0.233,0.600]
ST001705	Train	0.952<br>[0.952,0.952]	0.915<br>[0.915,0.915]	0.952<br>[0.952,0.952]	0.956<br>[0.956,0.956]	0.976<br>[0.976,0.976]
	Test	**0.966**<br>**[0.907,1.000]**	0.906<br>[0.831,0.970]	**0.966**<br>**[0.906,1.000]**	**0.966**<br>**[0.909,1.000]**	0.940<br>[0.868,1.000]
ST002498	Train	0.543<br>[0.543,0.543]	0.537<br>[0.537,0.537]	0.545<br>[0.545,0.545]	0.567<br>[0.567,0.567]	0.536<br>[0.536,0.536]
	Test	**0.583**<br>**[0.490,0.677]**	0.556<br>[0.475,0.635]	0.560<br>[0.468,0.651]	0.562<br>[0.477,0.640]	0.566<br>[0.477,0.655]

Dataset		BNN	CNN	FNN	KAN	SNN
ST002773	Train	0.460<br>[0.460,0.460]	0.442<br>[0.442,0.442]	0.454<br>[0.454,0.454]	0.511<br>[0.511,0.511]	0.444<br>[0.444,0.444]
	Test	0.411<br>[0.331,0.500]	0.467<br>[0.392,0.546]	0.419<br>[0.333,0.504]	**0.558**<br>**[0.447,0.659]**	0.454<br>[0.380,0.529]
ST003048	Train	0.924<br>[0.924,0.924]	0.879<br>[0.879,0.879]	0.927<br>[0.927,0.927]	0.900<br>[0.900,0.900]	0.923<br>[0.923,0.923]
	Test	0.886<br>[0.815,0.956]	0.845<br>[0.758,0.928]	0.923<br>[0.857,0.980]	**0.926**<br>**[0.857,0.984]**	0.919<br>[0.841,0.982]

Table S2. Recall and 95% CIs across network-based models and datasets. CIs for the training data were computed from the recalls of the 50 folds (derived from 5-fold stratified cross-validation with 10 different partitions). CIs for the test data were calculated using the bootstrap method with 10,000 resamplings. For each dataset, the largest recall on the test data across the network-based models is highlighted in bold. BNN: Bayesian Neural Network; CNN: Convolutional Neural Network; FNN: Feedforward Neural Network; KAN: Kolmogorov-Arnold Network; SNN: Spiking Neural Network.

Dataset		BNN	CNN	FNN	KAN	SNN
MTBLS136	Train	0.723<br>[0.723,0.723]	0.672<br>[0.672,0.672]	0.719<br>[0.719,0.719]	0.680<br>[0.680,0.680]	0.668<br>[0.668,0.668]
	Test	0.673<br>[0.587,0.766]	0.636<br>[0.539,0.722]	0.673<br>[0.588,0.752]	**0.691**<br>**[0.607,0.773]**	0.609<br>[0.519,0.701]
MTBLS161	Train	0.760<br>[0.760,0.760]	0.710<br>[0.710,0.710]	0.785<br>[0.785,0.785]	0.768<br>[0.768,0.768]	0.732<br>[0.732,0.732]
	Test	0.625<br>[0.250,1.000]	**0.750**<br>**[0.400,1.000]**	**0.750**<br>**[0.375,1.000]**	0.250<br>[0.000,0.600]	**0.750**<br>**[0.400,1.000]**
MTBLS404	Train	0.835<br>[0.835,0.835]	0.745<br>[0.745,0.745]	0.842<br>[0.842,0.842]	0.831<br>[0.831,0.831]	0.824<br>[0.824,0.824]
	Test	**0.857**<br>**[0.720,0.968]**	0.786<br>[0.600,0.926]	**0.857**<br>**[0.720,0.967]**	0.750<br>[0.591,0.903]	0.786<br>[0.630,0.929]
MTBLS547	Train	0.874<br>[0.874,0.874]	0.896<br>[0.896,0.896]	0.874<br>[0.874,0.874]	0.832<br>[0.832,0.832]	0.859<br>[0.859,0.859]

Dataset		BNN	CNN	FNN	KAN	SNN
	Test	**0.765 [0.545,0.941]**	**0.765 [0.550,0.947]**	**0.765 [0.538,0.944]**	**0.765 [0.533,0.944]**	**0.765 [0.555,0.944]**
MTBLS90	Train	0.750 [0.750,0.750]	0.721 [0.721,0.721]	0.775 [0.775,0.775]	0.713 [0.713,0.713]	0.742 [0.742,0.742]
	Test	0.621 [0.546,0.701]	0.801 [0.740,0.865]	0.801 [0.738,0.861]	**1.000 [1.000,1.000]**	0.696 [0.626,0.767]
MTBLS92	Train	0.814 [0.814,0.814]	0.784 [0.784,0.784]	0.840 [0.840,0.840]	0.815 [0.815,0.815]	0.795 [0.795,0.795]
	Test	**0.688 [0.545,0.816]**	**0.688 [0.545,0.821]**	**0.688 [0.558,0.811]**	**0.688 [0.549,0.818]**	**0.688 [0.547,0.820]**
ST000355	Train	0.968 [0.968,0.968]	0.862 [0.862,0.862]	0.946 [0.946,0.946]	0.904 [0.904,0.904]	0.940 [0.940,0.940]
	Test	**0.962 [0.864,1.000]**	0.808 [0.652,0.957]	0.846 [0.708,0.967]	**0.962 [0.875,1.000]**	**0.962 [0.864,1.000]**
ST000369	Train	0.636 [0.636,0.636]	0.472 [0.472,0.472]	0.700 [0.700,0.700]	0.527 [0.527,0.527]	0.560 [0.560,0.560]
	Test	0.800 [0.500,1.000]	**0.900 [0.667,1.000]**	0.800 [0.500,1.000]	0.500 [0.182,0.846]	**0.900 [0.667,1.000]**
ST000496	Train	0.910 [0.910,0.910]	0.864 [0.864,0.864]	0.905 [0.905,0.905]	0.863 [0.863,0.863]	0.878 [0.878,0.878]
	Test	**0.941 [0.800,1.000]**	0.765 [0.545,0.941]	**0.941 [0.812,1.000]**	**0.941 [0.800,1.000]**	0.824 [0.625,1.000]
ST001000	Train	0.634 [0.634,0.634]	0.611 [0.611,0.611]	0.683 [0.683,0.683]	0.663 [0.663,0.663]	0.700 [0.700,0.700]
	Test	0.389 [0.167,0.625]	0.389 [0.167,0.615]	0.444 [0.231,0.682]	**0.722 [0.500,0.923]**	0.556 [0.312,0.789]
ST001047	Train	0.887 [0.887,0.887]	0.786 [0.786,0.786]	0.864 [0.864,0.864]	0.812 [0.812,0.812]	0.839 [0.839,0.839]
	Test	**0.923 [0.733,1.000]**	**0.923 [0.750,1.000]**	0.769 [0.500,1.000]	0.769 [0.545,1.000]	0.846 [0.636,1.000]
ST001082	Train	0.969 [0.969,0.969]	0.971 [0.971,0.971]	0.973 [0.973,0.973]	0.797 [0.797,0.797]	0.873 [0.873,0.873]
	Test	**0.978 [0.942,1.000]**	**0.978 [0.944,1.000]**	0.956 [0.911,0.989]	0.533 [0.429,0.639]	0.933 [0.875,0.978]

Dataset		BNN	CNN	FNN	KAN	SNN
ST001682	Train	0.400 [0.400,0.400]	0.411 [0.411,0.411]	0.424 [0.424,0.424]	0.410 [0.410,0.410]	0.403 [0.403,0.403]
	Test	0.350 [0.205,0.500]	0.450 [0.289,0.595]	0.400 [0.244,0.553]	**0.600 [0.439,0.744]**	0.300 [0.160,0.450]
ST001705	Train	0.923 [0.923,0.923]	0.978 [0.978,0.978]	0.951 [0.951,0.951]	0.962 [0.962,0.962]	0.889 [0.889,0.889]
	Test	0.966 [0.914,1.000]	**1.000 [1.000,1.000]**	0.983 [0.939,1.000]	0.966 [0.911,1.000]	0.810 [0.698,0.909]
ST002498	Train	0.524 [0.524,0.524]	0.597 [0.597,0.597]	0.559 [0.559,0.559]	0.602 [0.602,0.602]	0.537 [0.537,0.537]
	Test	0.600 [0.509,0.685]	**0.762 [0.679,0.840]**	0.619 [0.524,0.712]	0.695 [0.612,0.781]	0.571 [0.475,0.670]
ST002773	Train	0.467 [0.467,0.467]	0.467 [0.467,0.467]	0.495 [0.495,0.495]	0.996 [0.996,0.996]	0.448 [0.448,0.448]
	Test	0.371 [0.293,0.457]	0.545 [0.465,0.628]	0.399 [0.321,0.477]	0.336 [0.258,0.412]	**0.552 [0.473,0.630]**
ST003048	Train	0.902 [0.902,0.902]	0.880 [0.880,0.880]	0.921 [0.921,0.921]	0.896 [0.896,0.896]	0.892 [0.892,0.892]
	Test	0.912 [0.843,0.971]	0.882 [0.803,0.955]	0.882 [0.803,0.954]	**0.926 [0.857,0.981]**	0.838 [0.743,0.919]

Table S3. Sensitivity and 95% CIs across network-based models and datasets. CIs for the training data were computed from the sensitivities of the 50 folds (derived from 5-fold stratified cross-validation with 10 different partitions). CIs for the test data were calculated using the bootstrap method with 10,000 resamplings. For each dataset, the largest sensitivity on the test data across the network-based models is highlighted in bold. BNN: Bayesian Neural Network; CNN: Convolutional Neural Network; FNN: Feedforward Neural Network; KAN: Kolmogorov-Arnold Network; SNN: Spiking Neural Network.

Dataset		BNN	CNN	FNN	KAN	SNN
MTBLS136	Train	0.723 [0.723,0.723]	0.672 [0.672,0.672]	0.719 [0.719,0.719]	0.680 [0.680,0.680]	0.668 [0.668,0.668]

Dataset		BNN	CNN	FNN	KAN	SNN
	Test	0.673 [0.579,0.756]	0.636 [0.546,0.725]	0.673 [0.583,0.760]	**0.691 [0.608,0.781]**	0.609 [0.522,0.701]
MTBLS161	Train	0.760 [0.760,0.760]	0.710 [0.710,0.710]	0.785 [0.785,0.785]	0.768 [0.768,0.768]	0.732 [0.732,0.732]
	Test	0.625 [0.250,1.000]	**0.750 [0.400,1.000]**	**0.750 [0.400,1.000]**	0.250 [0.000,0.600]	**0.750 [0.400,1.000]**
MTBLS404	Train	0.835 [0.835,0.835]	0.745 [0.745,0.745]	0.842 [0.842,0.842]	0.831 [0.831,0.831]	0.824 [0.824,0.824]
	Test	**0.857 [0.708,0.967]**	0.786 [0.609,0.929]	**0.857 [0.720,0.968]**	0.750 [0.571,0.903]	0.786 [0.625,0.926]
MTBLS547	Train	0.874 [0.874,0.874]	0.896 [0.896,0.896]	0.874 [0.874,0.874]	0.832 [0.832,0.832]	0.859 [0.859,0.859]
	Test	**0.765 [0.538,0.944]**	**0.765 [0.545,0.944]**	**0.765 [0.533,0.941]**	**0.765 [0.562,0.941]**	**0.765 [0.556,0.941]**
MTBLS90	Train	0.750 [0.750,0.750]	0.721 [0.721,0.721]	0.775 [0.775,0.775]	0.713 [0.713,0.713]	0.742 [0.742,0.742]
	Test	0.621 [0.544,0.696]	0.801 [0.739,0.863]	0.801 [0.740,0.863]	**1.000 [1.000,1.000]**	0.696 [0.624,0.766]
MTBLS92	Train	0.814 [0.814,0.814]	0.784 [0.784,0.784]	0.840 [0.840,0.840]	0.815 [0.815,0.815]	0.795 [0.795,0.795]
	Test	**0.688 [0.547,0.812]**	**0.688 [0.548,0.824]**	**0.688 [0.551,0.813]**	**0.688 [0.548,0.826]**	**0.688 [0.558,0.813]**
ST000355	Train	0.968 [0.968,0.968]	0.862 [0.862,0.862]	0.946 [0.946,0.946]	0.904 [0.904,0.904]	0.940 [0.940,0.940]
	Test	**0.962 [0.870,1.000]**	0.808 [0.656,0.955]	0.846 [0.692,0.964]	**0.962 [0.870,1.000]**	**0.962 [0.880,1.000]**
ST000369	Train	0.636 [0.636,0.636]	0.472 [0.472,0.472]	0.700 [0.700,0.700]	0.527 [0.527,0.527]	0.560 [0.560,0.560]
	Test	0.800 [0.500,1.000]	**0.900 [0.636,1.000]**	0.800 [0.500,1.000]	0.500 [0.167,0.800]	**0.900 [0.667,1.000]**
ST000496	Train	0.910 [0.910,0.910]	0.864 [0.864,0.864]	0.905 [0.905,0.905]	0.863 [0.863,0.863]	0.878 [0.878,0.878]
	Test	**0.941 [0.800,1.000]**	0.765 [0.550,0.944]	**0.941 [0.812,1.000]**	**0.941 [0.812,1.000]**	0.824 [0.636,1.000]

Dataset		BNN	CNN	FNN	KAN	SNN
ST001000	Train	0.634 [0.634,0.634]	0.611 [0.611,0.611]	0.683 [0.683,0.683]	0.663 [0.663,0.663]	0.700 [0.700,0.700]
	Test	0.389 [0.176,0.600]	0.389 [0.176,0.625]	0.444 [0.214,0.684]	**0.722 [0.500,0.929]**	0.556 [0.333,0.786]
ST001047	Train	0.887 [0.887,0.887]	0.786 [0.786,0.786]	0.864 [0.864,0.864]	0.812 [0.812,0.812]	0.839 [0.839,0.839]
	Test	**0.923 [0.750,1.000]**	**0.923 [0.750,1.000]**	0.769 [0.533,1.000]	0.769 [0.500,1.000]	0.846 [0.615,1.000]
ST001082	Train	0.969 [0.969,0.969]	0.971 [0.971,0.971]	0.973 [0.973,0.973]	0.797 [0.797,0.797]	0.873 [0.873,0.873]
	Test	**0.978 [0.941,1.000]**	**0.978 [0.943,1.000]**	0.956 [0.909,0.990]	0.533 [0.427,0.636]	0.933 [0.877,0.978]
ST001682	Train	0.400 [0.400,0.400]	0.411 [0.411,0.411]	0.424 [0.424,0.424]	0.410 [0.410,0.410]	0.403 [0.403,0.403]
	Test	0.350 [0.206,0.500]	0.450 [0.308,0.600]	0.400 [0.250,0.561]	**0.600 [0.432,0.744]**	0.300 [0.162,0.444]
ST001705	Train	0.923 [0.923,0.923]	0.978 [0.978,0.978]	0.951 [0.951,0.951]	0.962 [0.962,0.962]	0.889 [0.889,0.889]
	Test	0.966 [0.912,1.000]	**1.000 [1.000,1.000]**	0.983 [0.944,1.000]	0.966 [0.912,1.000]	0.810 [0.702,0.907]
ST002498	Train	0.524 [0.524,0.524]	0.597 [0.597,0.597]	0.559 [0.559,0.559]	0.602 [0.602,0.602]	0.537 [0.537,0.537]
	Test	0.600 [0.505,0.689]	**0.762 [0.681,0.838]**	0.619 [0.529,0.709]	0.695 [0.610,0.775]	0.571 [0.474,0.664]
ST002773	Train	0.467 [0.467,0.467]	0.467 [0.467,0.467]	0.495 [0.495,0.495]	0.996 [0.996,0.996]	0.448 [0.448,0.448]
	Test	0.371 [0.296,0.453]	0.545 [0.461,0.629]	0.399 [0.321,0.485]	0.336 [0.268,0.412]	**0.552 [0.464,0.631]**
ST003048	Train	0.902 [0.902,0.902]	0.880 [0.880,0.880]	0.921 [0.921,0.921]	0.896 [0.896,0.896]	0.892 [0.892,0.892]
	Test	0.912 [0.841,0.971]	0.882 [0.797,0.957]	0.882 [0.803,0.958]	**0.926 [0.864,0.985]**	0.838 [0.750,0.918]

Table S4. Specificity and 95% CIs across network-based models and datasets. CIs for the training data were computed from the specificities of the 50 folds (derived from 5-fold stratified cross-validation with 10 different partitions). CIs for the test data were calculated using the bootstrap method with 10,000 resamplings. For each dataset, the largest specificity on the test data across the network-based models is highlighted in bold. BNN: Bayesian Neural Network; CNN: Convolutional Neural Network; FNN: Feedforward Neural Network; KAN: Kolmogorov-Arnold Network; SNN: Spiking Neural Network.

Dataset		BNN	CNN	FNN	KAN	SNN
MTBLS136	Train	0.712 [0.712,0.712]	0.681 [0.681,0.681]	0.708 [0.708,0.708]	0.685 [0.685,0.685]	0.738 [0.738,0.738]
	Test	**0.752 [0.664,0.828]**	0.743 [0.667,0.819]	0.681 [0.602,0.764]	0.717 [0.630,0.800]	0.699 [0.617,0.785]
MTBLS161	Train	0.700 [0.700,0.700]	0.766 [0.766,0.766]	0.733 [0.733,0.733]	0.755 [0.755,0.755]	0.683 [0.683,0.683]
	Test	**1.000 [1.000,1.000]**	0.833 [0.600,1.000]	**1.000 [1.000,1.000]**	0.833 [0.600,1.000]	0.917 [0.727,1.000]
MTBLS404	Train	0.902 [0.902,0.902]	0.821 [0.821,0.821]	0.910 [0.910,0.910]	0.895 [0.895,0.895]	0.857 [0.857,0.857]
	Test	**0.794 [0.647,0.920]**	0.706 [0.552,0.852]	0.735 [0.576,0.871]	0.735 [0.586,0.875]	0.765 [0.606,0.902]
MTBLS547	Train	0.923 [0.923,0.923]	0.900 [0.900,0.900]	0.920 [0.920,0.920]	0.880 [0.880,0.880]	0.920 [0.920,0.920]
	Test	**0.875 [0.688,1.000]**	**0.875 [0.688,1.000]**	0.812 [0.591,1.000]	0.812 [0.600,1.000]	**0.875 [0.706,1.000]**
MTBLS90	Train	0.758 [0.758,0.758]	0.702 [0.702,0.702]	0.775 [0.775,0.775]	0.712 [0.712,0.712]	0.767 [0.767,0.767]
	Test	**0.852 [0.796,0.906]**	0.759 [0.692,0.824]	0.815 [0.756,0.874]	0.000 [0.000,0.000]	0.833 [0.772,0.885]
MTBLS92	Train	0.711 [0.711,0.711]	0.674 [0.674,0.674]	0.687 [0.687,0.687]	0.752 [0.752,0.752]	0.699 [0.699,0.699]
	Test	0.703 [0.553,0.842]	0.541 [0.371,0.700]	**0.730 [0.571,0.857]**	0.595 [0.441,0.744]	**0.730 [0.576,0.868]**
ST000355	Train	0.961 [0.961,0.961]	0.972 [0.972,0.972]	0.986 [0.986,0.986]	0.981 [0.981,0.981]	0.979 [0.979,0.979]
	Test	0.913 [0.829,0.979]	0.935 [0.851,1.000]	0.935 [0.857,1.000]	**0.978 [0.927,1.000]**	0.957 [0.889,1.000]

Dataset		BNN	CNN	FNN	KAN	SNN
ST000369	Train	0.662 [0.662,0.662]	0.769 [0.769,0.769]	0.570 [0.570,0.570]	0.761 [0.761,0.761]	0.682 [0.682,0.682]
	Test	0.588 [0.357,0.824]	**0.824 [0.636,1.000]**	0.765 [0.533,0.941]	0.647 [0.389,0.850]	**0.824 [0.625,1.000]**
ST000496	Train	0.865 [0.865,0.865]	0.782 [0.782,0.782]	0.870 [0.870,0.870]	0.784 [0.784,0.784]	0.838 [0.838,0.838]
	Test	**0.882 [0.688,1.000]**	0.706 [0.471,0.917]	**0.882 [0.706,1.000]**	0.765 [0.533,0.941]	**0.882 [0.706,1.000]**
ST001000	Train	0.762 [0.762,0.762]	0.784 [0.784,0.784]	0.740 [0.740,0.740]	0.738 [0.738,0.738]	0.729 [0.729,0.729]
	Test	0.696 [0.500,0.889]	**0.826 [0.640,0.962]**	0.739 [0.555,0.913]	0.609 [0.389,0.824]	0.739 [0.545,0.909]
ST001047	Train	0.837 [0.837,0.837]	0.767 [0.767,0.767]	0.832 [0.832,0.832]	0.840 [0.840,0.840]	0.852 [0.852,0.852]
	Test	0.667 [0.400,0.909]	**0.933 [0.778,1.000]**	0.733 [0.500,0.938]	0.733 [0.526,0.933]	0.667 [0.375,0.889]
ST001082	Train	0.966 [0.966,0.966]	0.960 [0.960,0.960]	0.964 [0.964,0.964]	0.221 [0.221,0.221]	0.933 [0.933,0.933]
	Test	0.987 [0.955,1.000]	0.974 [0.933,1.000]	**1.000 [1.000,1.000]**	0.500 [0.390,0.613]	0.974 [0.934,1.000]
ST001682	Train	0.474 [0.474,0.474]	0.459 [0.459,0.459]	0.460 [0.460,0.460]	0.484 [0.484,0.484]	0.480 [0.480,0.480]
	Test	0.372 [0.225,0.513]	0.372 [0.234,0.512]	0.488 [0.341,0.630]	0.488 [0.346,0.638]	**0.605 [0.457,0.756]**
ST001705	Train	0.895 [0.895,0.895]	0.789 [0.789,0.789]	0.895 [0.895,0.895]	0.899 [0.899,0.899]	0.952 [0.952,0.952]
	Test	**0.929 [0.818,1.000]**	0.786 [0.630,0.926]	**0.929 [0.818,1.000]**	**0.929 [0.818,1.000]**	0.893 [0.762,1.000]
ST002498	Train	0.486 [0.486,0.486]	0.401 [0.401,0.401]	0.457 [0.457,0.457]	0.461 [0.461,0.461]	0.461 [0.461,0.461]
	Test	**0.494 [0.400,0.595]**	0.281 [0.193,0.374]	0.427 [0.329,0.527]	0.360 [0.261,0.462]	0.483 [0.378,0.584]
ST002773	Train	0.432 [0.432,0.432]	0.396 [0.396,0.396]	0.378 [0.378,0.378]	0.005 [0.005,0.005]	0.420 [0.420,0.420]

Dataset		BNN	CNN	FNN	KAN	SNN
	Test	0.445<br>[0.369,0.534]	0.350<br>[0.274,0.430]	0.423<br>[0.340,0.511]	**0.723**<br>**[0.646,0.800]**	0.307<br>[0.235,0.387]
ST003048	Train	0.918<br>[0.918,0.918]	0.866<br>[0.866,0.866]	0.920<br>[0.920,0.920]	0.890<br>[0.890,0.890]	0.917<br>[0.917,0.917]
	Test	0.875<br>[0.794,0.952]	0.828<br>[0.734,0.925]	**0.922**<br>**[0.843,0.984]**	**0.922**<br>**[0.850,0.984]**	**0.922**<br>**[0.852,0.985]**
