# Supplementary figures and images for: A Comparative Study of Network-Based Machine Learning Approaches for Binary Classification in Metabolomics
Source: Metabolites. 2025 Mar 3;15(3):174. doi: 10.3390/metabo15030174 (PMC11944042; doi:10.3390/metabo15030174)

MTBLS136

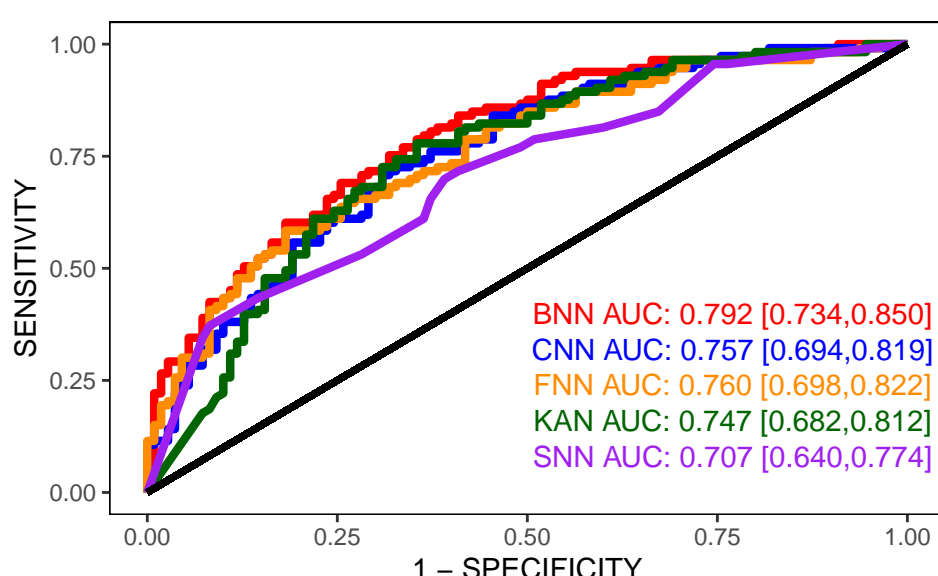

MTBLS161

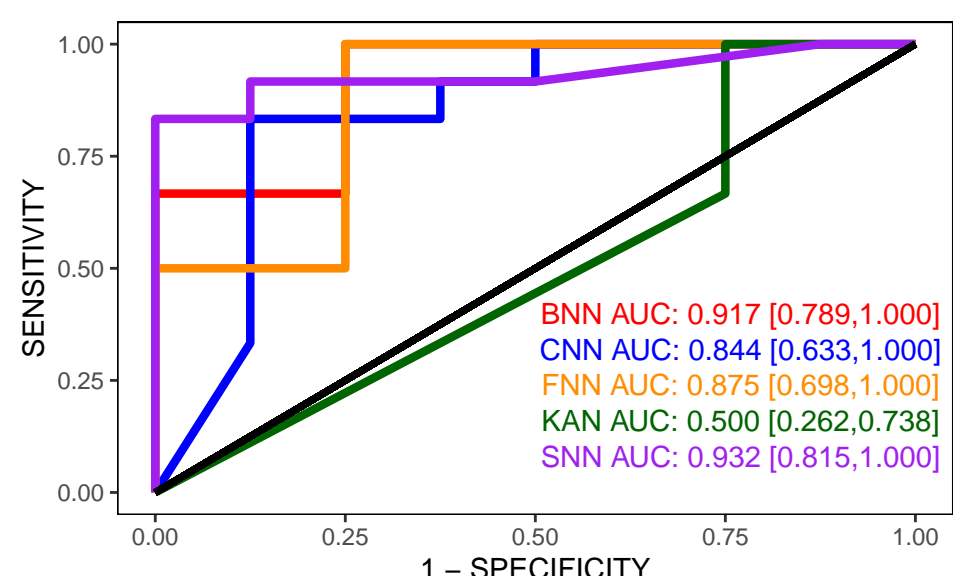

MTBLS404

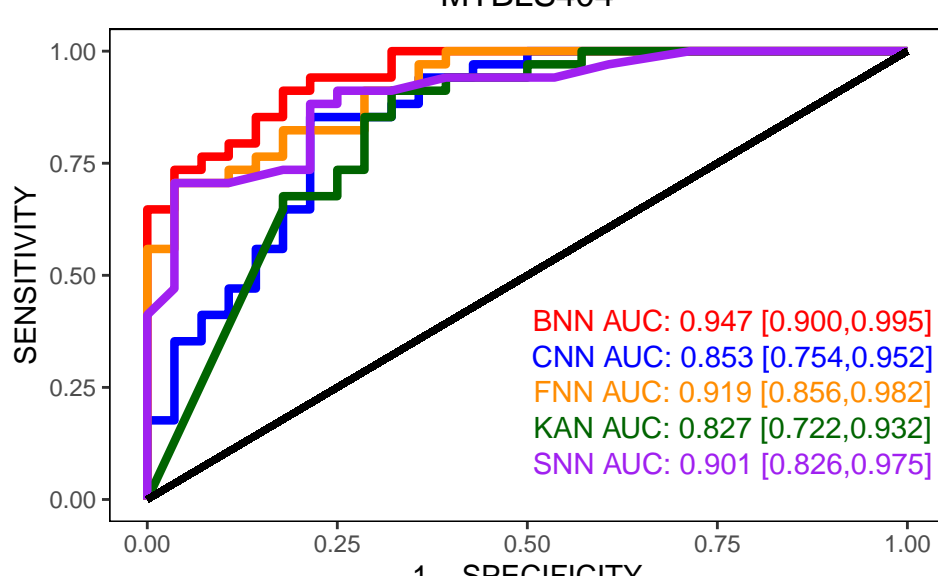

MTBLS547

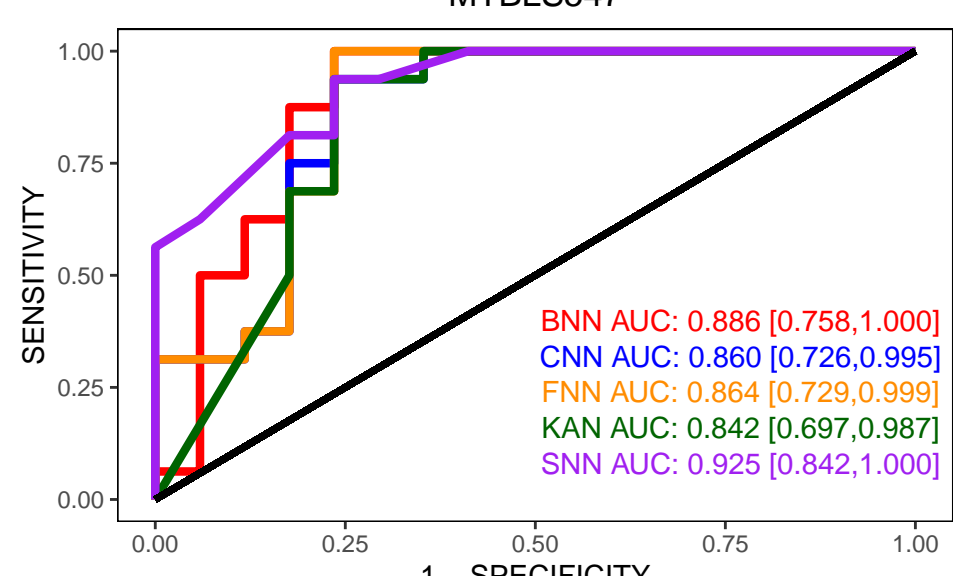

MTBLS90

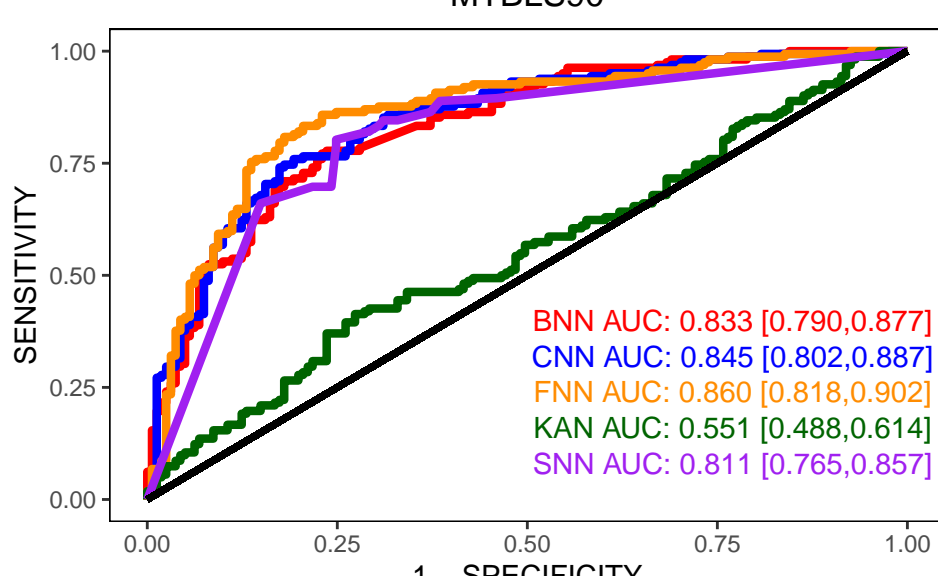

MTBLS92

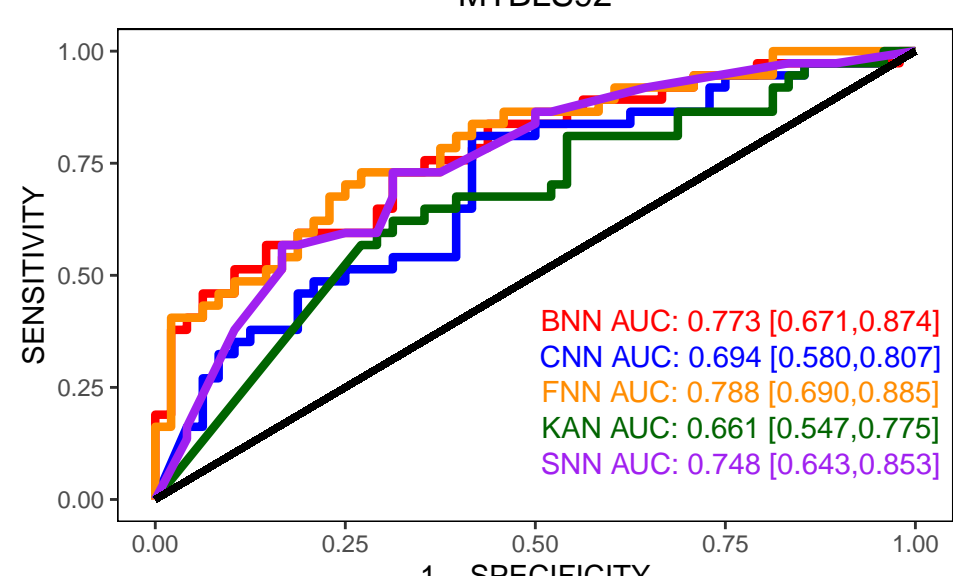

ST000355

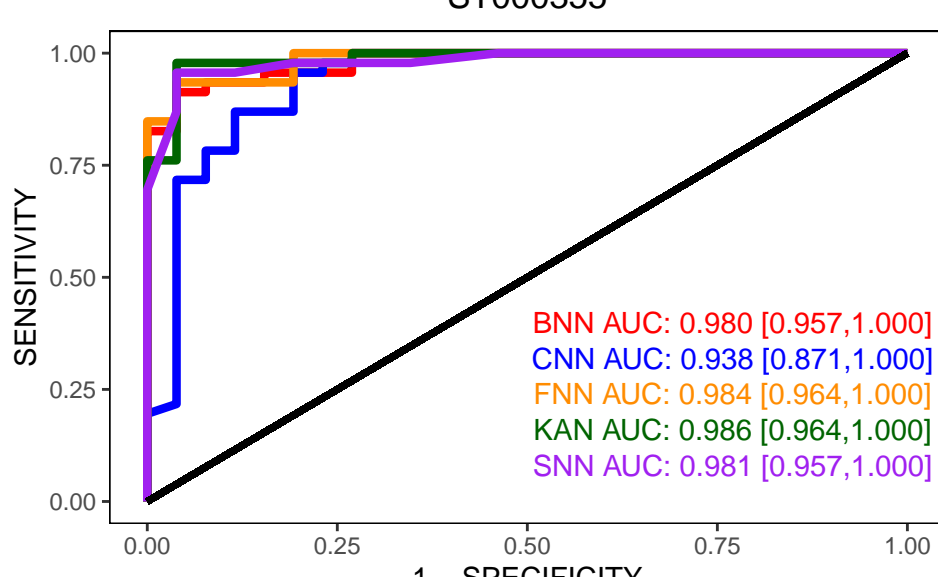

ST000369

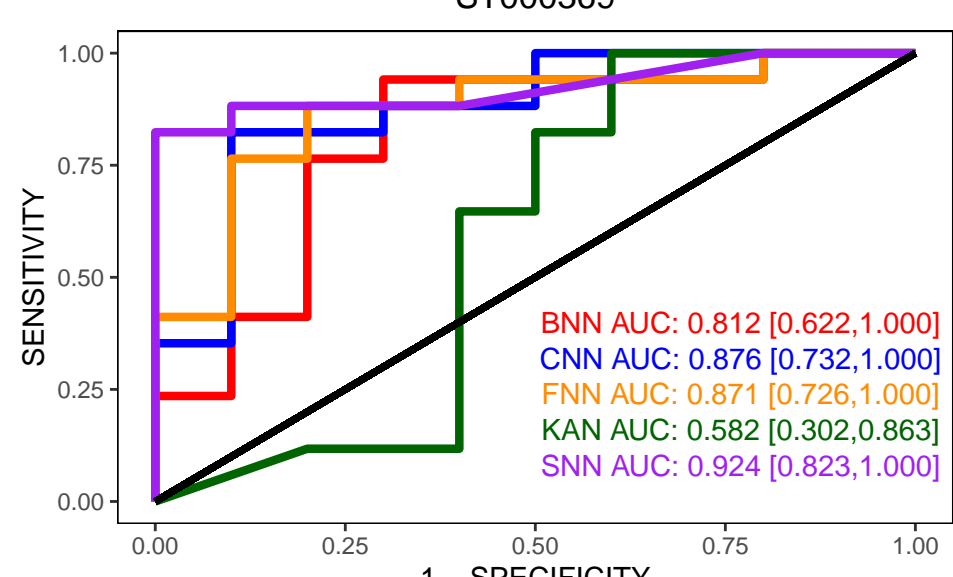

ST000496

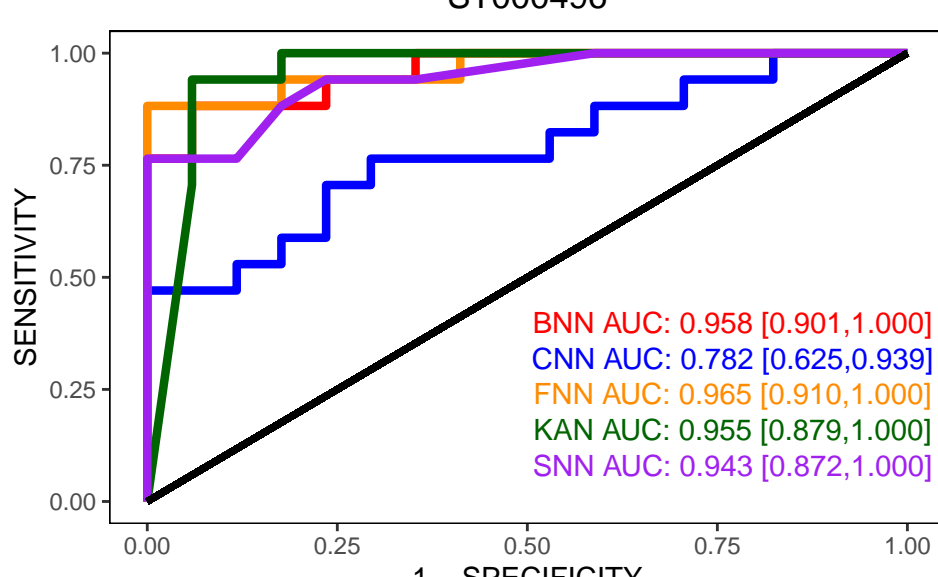

ST001000

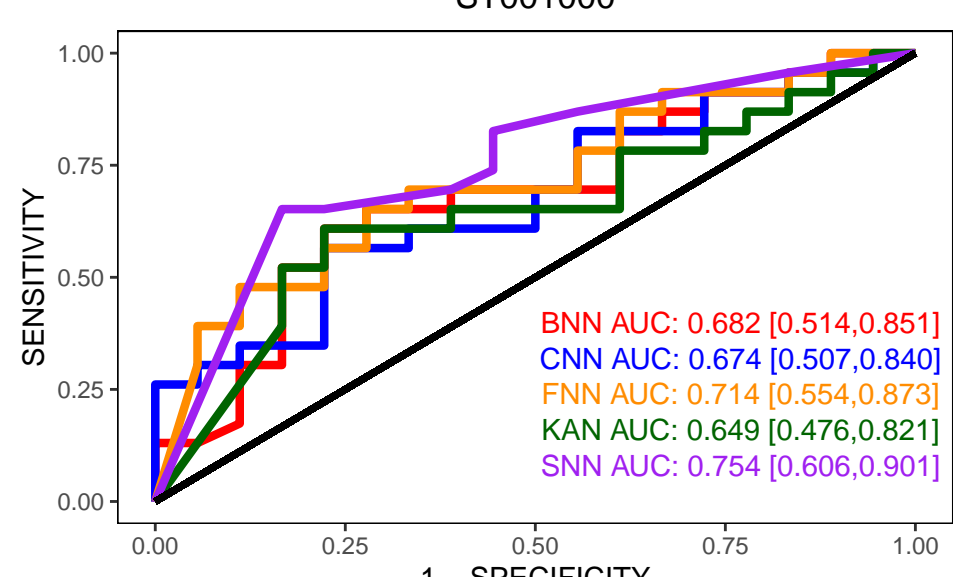

ST001047

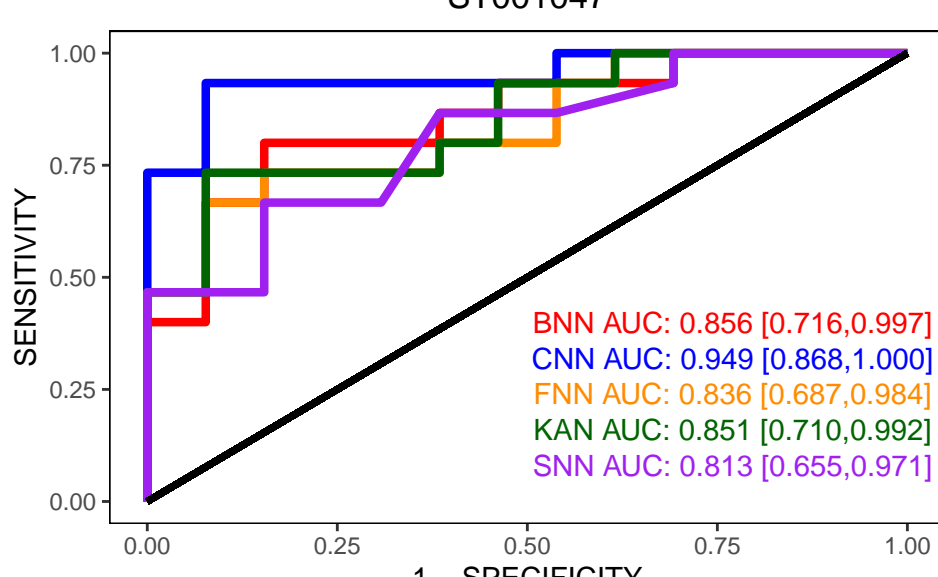

ST001082

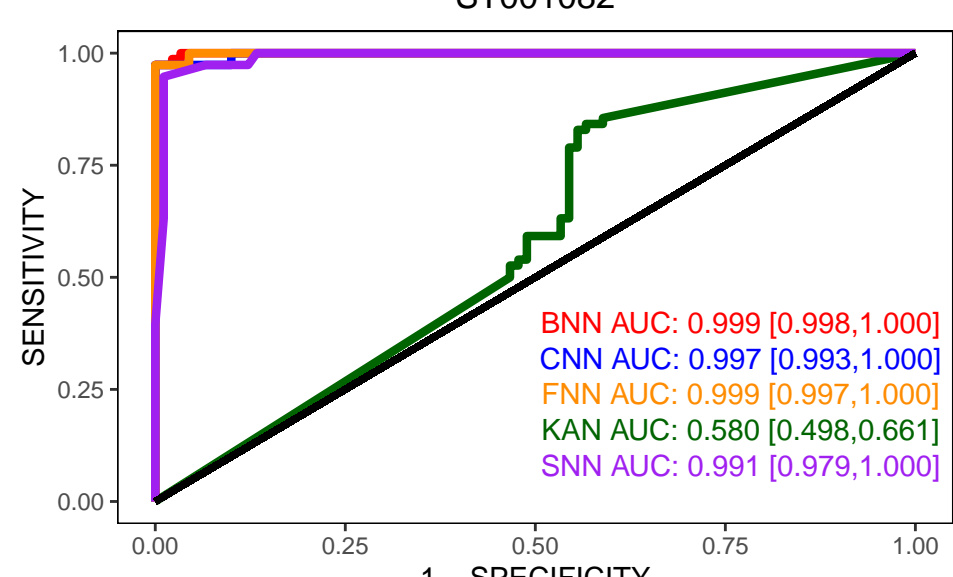

ST001682

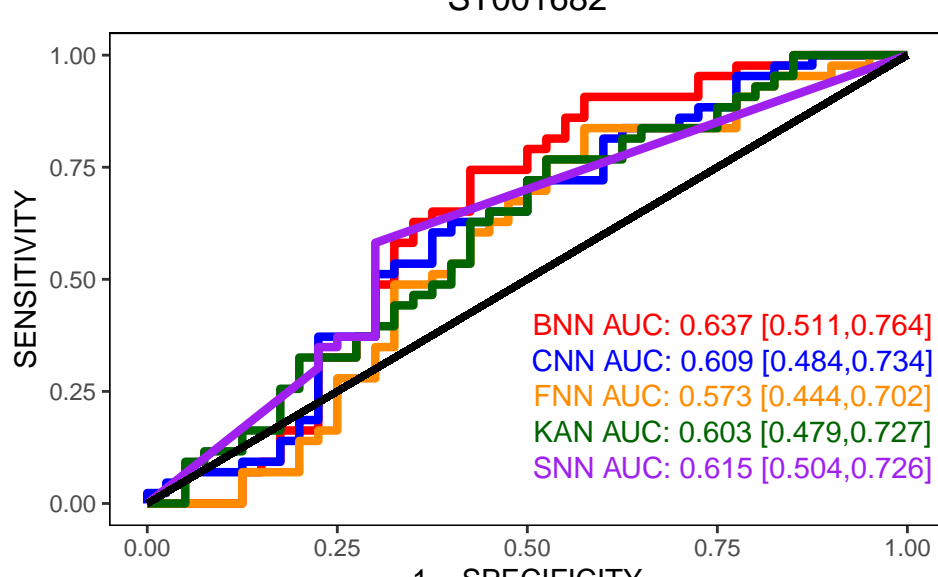

ST001705

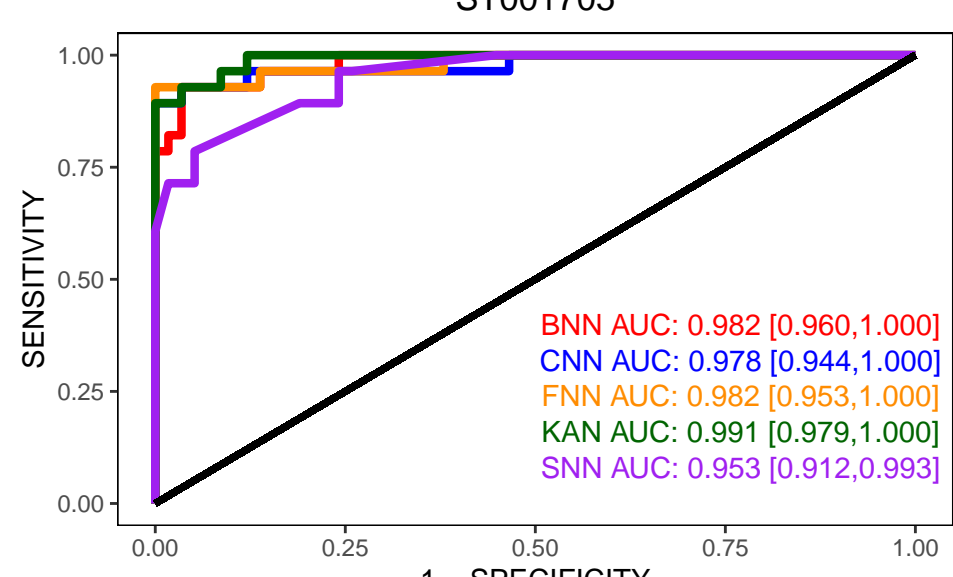

ST002498

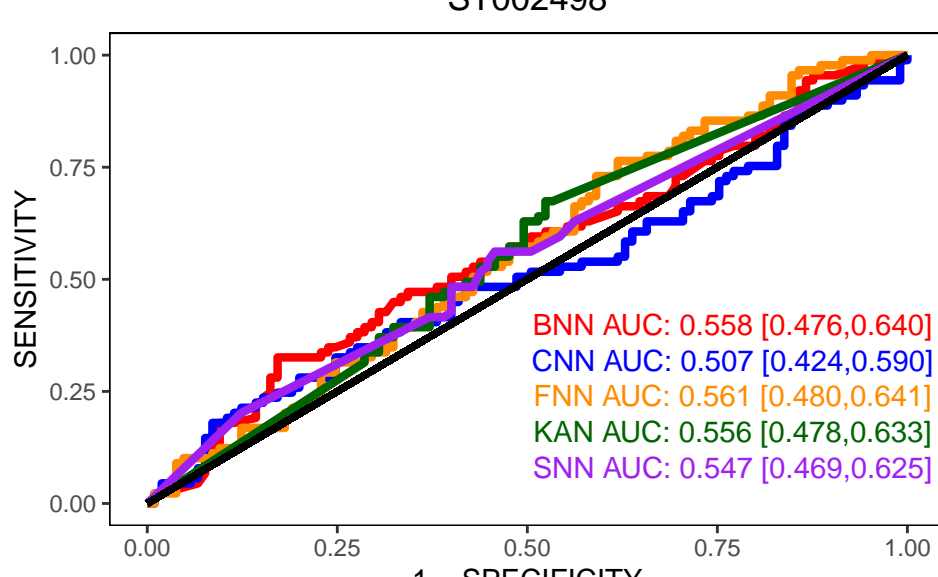

ST002773

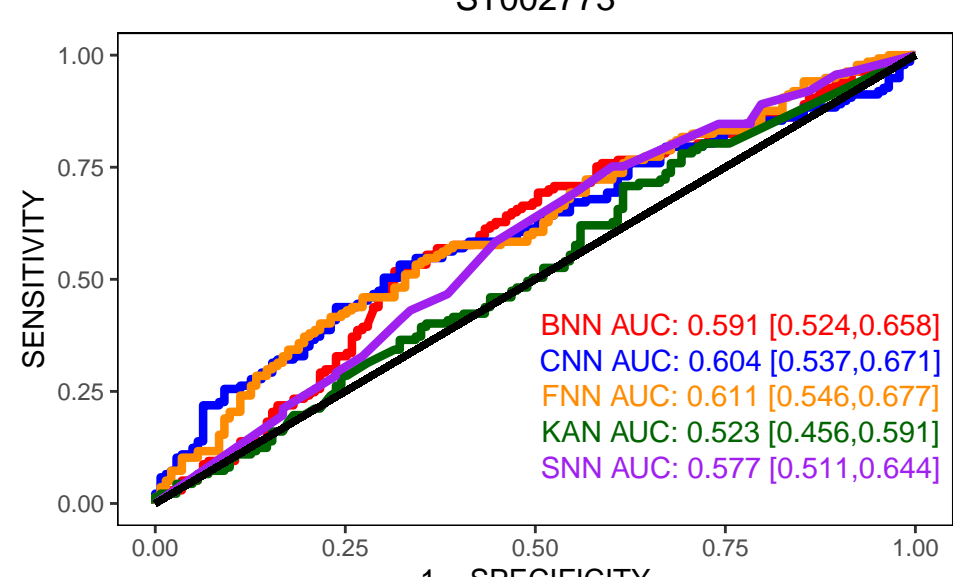

ST003048

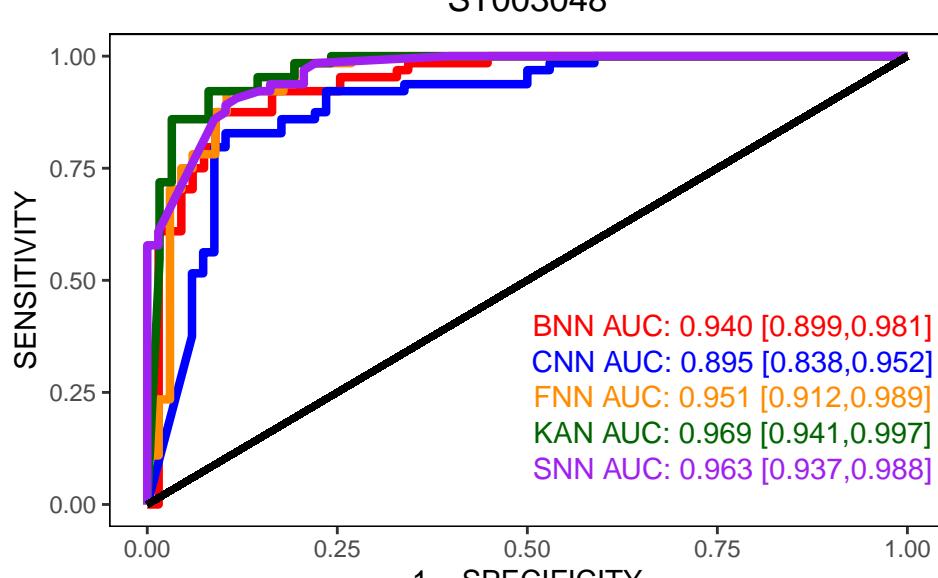

Model

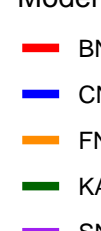

Supplement: Supplementary file 1 [file metabolites-15-00174-s001.zip › supplementary/S1.pdf]
